# Supplementary material for: ST2/IL-33 axis blockade inhibits regulatory T cell cytotoxicity towards CD8 T cells in the leukemic niche
Source: Nat Commun. 2025 Jul 21;16:6580. doi: 10.1038/s41467-025-61647-8 (PMC12279971; doi:10.1038/s41467-025-61647-8)
Supplement: Supplementary file 1 — Supplementary Information [file 41467_2025_61647_MOESM1_ESM.pdf]

ST2/IL-33 axis blockade inhibits regulatory T cell cytotoxicity towards CD8 T cells in the leukemic niche

Hua Jiang[1,2&], Denggang Fu[1,2&], Santhosh Kumar Pasupuleti[2], Baskar Ramdas[2], Alan Long[3], Abdulraouf M. Ramadan[2], Jinfeng Yang[2], Ramesh Kumar[2], Jessica H. Hartman[4], B Jacob Kendrick[5], Ed Simpson[6], Hongyu Gao[6], Yunlong Liu[6], Drew Moore[7], Suganya Subramanian[7], Stefano Berto[7], Anilkumar Gopalakrishnapillai[8], Sonali P. Barwe[8], Hongfen Guo[3], Nai-Kong V. Cheung[3], Reuben Kapur[2], Sophie Paczesny[1,2]

Supplementary Information (SI)

1. Supplementary Figures

Supplementary Fig. 1 Total T_{reg} cells and $ST2^+$ T_{reg} cells in healthy peripheral blood, and bone marrow in human and mice.

Supplementary Fig. 2 Tumor-infiltrating immune cells show differential Il1rl1 expression in four independent datasets of patients' AML.

Supplementary Fig. 3 Single cell RNA sequencing in BM from AML refractory and responder patients.

Supplementary Fig. 4 Gating strategies of flow cytometry analysis.

Supplementary Fig. 5 $ST2^+$ T_{reg} cells and total T_{reg} cells in BM from AML patients.

Supplementary Fig. 6 Frequencies of total $ST2^+$ T_{reg} cells post MLL-AF9 or DNMT3A/FLT3^{ITD}-mutant leukemic cell challenge.

Supplementary Fig. 7 Comparisons of percentages of T_{reg} cells positive for the indicated markers between paired $ST2^+$ T_{reg} cells and $ST2^-$ T_{reg} cells in the DNMT3A/FLT3^{ITD}-mutant model.

Supplementary Fig. 8 Correlation analysis of indicated CD8 subsets with $ST2^+$ T_{reg} cells in the DNMT3A/FLT3^{ITD}-mutant model.

Supplementary Fig. 9 Correlation analysis of $CD4^+$ T effector cells and NK cells with $ST2^+$ T_{reg} cells.

Supplementary Fig. 10 Statistically analyzed frequencies of total T_{reg} cells and activated T_{reg} cells in the adoptive transfer MLL-AF9 leukemia model.

Supplementary Fig. 11 Frequencies of MLL-AF9^{eGFP} cells in the malignant BM niches on day 10 post 10^6 leukemic cells challenge.

Supplementary Fig. 12 Number of CD8 T cells and frequencies of proliferated CD8 T cells post MLL-AF9^{eGFP} leukemic cells challenge.

Supplementary Fig. 13 WT-1 expression on murine MLL-AF9.

Supplementary Fig. 14 NanoString analysis of naive T_{reg} cells sorted from Tbet$^{-/-}$ and WT mice and frequencies of $ST2^+$ and total T_{reg} cells gated in T_{reg} cells sorted from Tbet$^{-/-}$ and WT mice.

Supplementary Fig. 15 Functional phenotypes of BM-derived $ST2^+$ TME (WT) T_{reg} cells distinguishing among ST2-expressing versus ST2-negative cells.

Supplementary Fig. 16 Statistical analysis of different CD8 subsets in mice with or without MLL-AF9 leukemia transferred with 20% WT or $ST2^{-/-}$ or $Tbet^{-/-}$ T_{reg} cells.

Supplementary Fig. 17 Statistically analyzed direct killing of $CD4^+$ T effector cells and NK cells by T_{reg} cells to using flow imaging.

Supplementary Fig. 18 HPLC, SPR and PK study of IgG-281 and IgG-282.

Supplementary Fig. 19 *In vitro* apoptosis and proliferation of TME-derived CD8 T treated with increasing IgG-281 concentrations.

Supplementary Fig. 20 Frequencies of immune cells parameters in the malignant BM niches on day 21 after the first administration of IgG control versus IgG-281+ anti-PD-1.

Supplementary Fig. 21 Anti-ST2 antibody promotes the abatement of $ST2^+T_{reg}$ cells to extend survival in HLA-A2-matched humanized AML model.

2. Supplementary Tables

Supplementary Table 1 AML patients' demographics.

Supplementary Table 2 Nanostring selected significant transcripts of BM-derived WT T_{reg} cells and $Tbet^{-/-}$ T_{reg} cells sorted from sex- and age-matched normal WT and $Tbet^{-/-}$ mice.

Supplementary Table 3 Selected genes represented in the heatmap of T_{reg} cells sorted from no leukemia cells transferred and malignant BM of mice in which ST2-/- T_{reg} cells versus WT (ST2+) T_{reg} cells versus Tbet-/- T_{reg} cells transferred.

Supplementary Table 4 Antibodies used for the flow cytometry analyses.

Supplementary Table 5 ChIP-qPCR Primers.

Supplementary Fig. 1 Total T_{reg} cells and ST2^{+} T_{reg} cells in healthy peripheral blood, and bone marrow in both human and mice

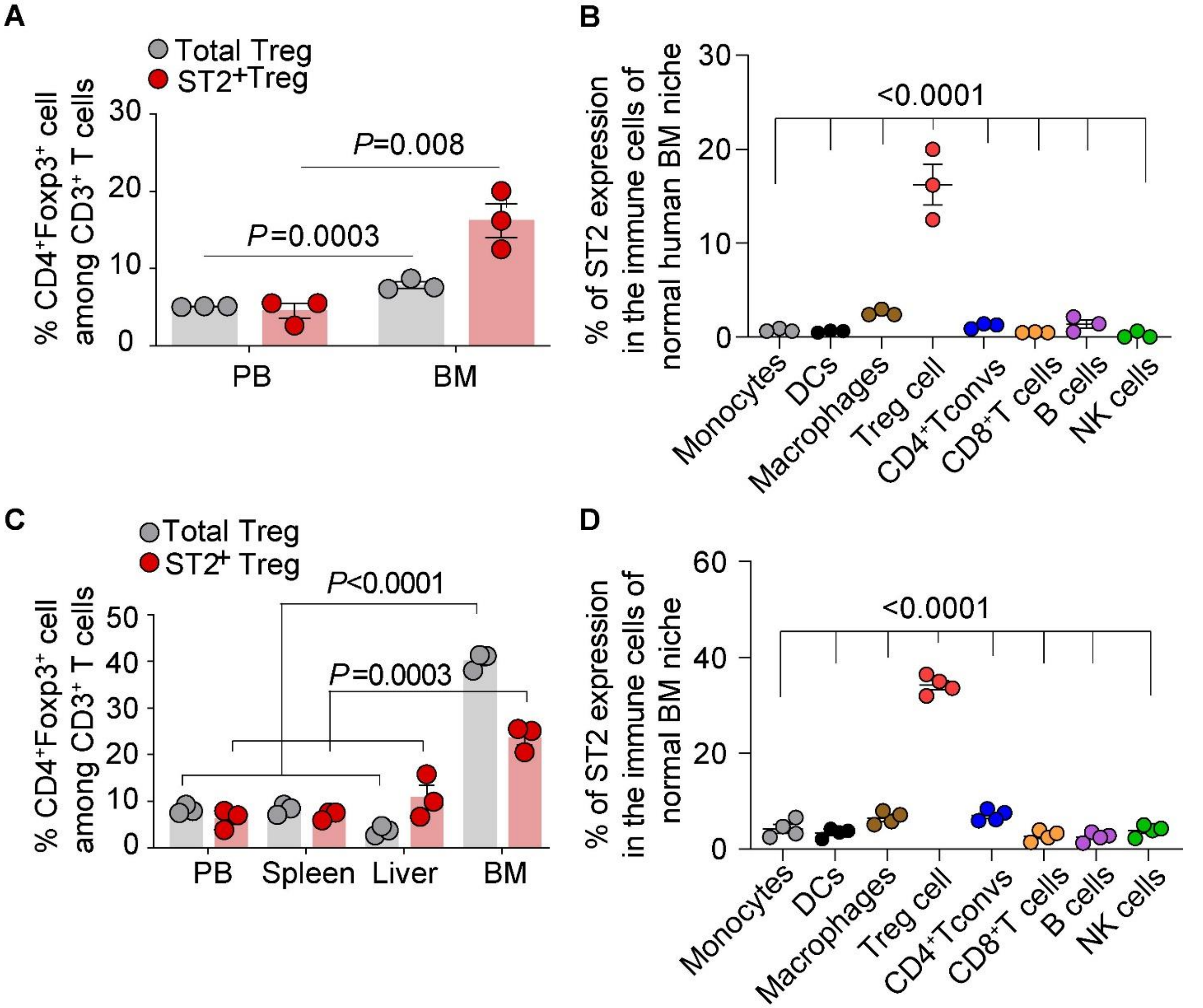

A, Comparison of the frequencies of both total T_{reg} cells and ST2^{+} T_{reg} cells among CD3^{+} T cells in peripheral blood and BM in the steady state in healthy human donors.
B, Frequencies of ST2 expression on immune cells in the normal human BM niche among total CD45^{+} cells. Graphed data represent two independent experiments (n=3).
C, Comparison of the frequencies of both total T_{reg} cells and ST2^{+} T_{reg} cells among CD3^{+} T cells in peripheral blood, secondary lymphoid organs (spleen, liver) and the BM in the steady state in healthy mice.
D, Frequencies of ST2 expression on immune cells in the normal BM niche among total CD45^{+} cells in healthy mice (n=4).
Unless specified otherwise, the data are presented as means ± s.e.m. (error bar) and compared using two-sided Student’s *t* test. Source data are provided as a Source Data file.

.

Supplementary Fig. 2 Tumor-infiltrating immune cells show differential Il1rl1 expression in four independent datasets of patients with AML

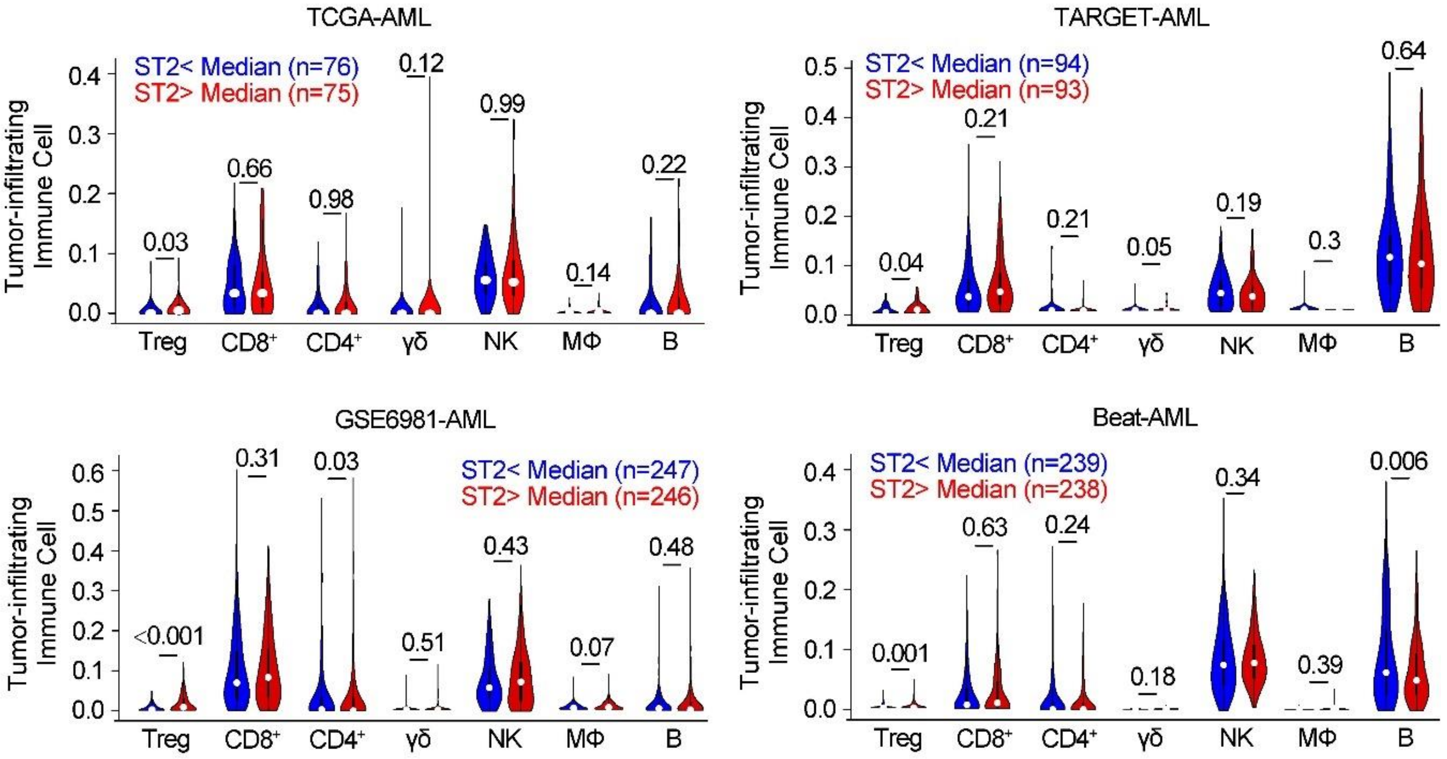

TCGA-AML: n=151; TARGET-AML: n=187; GSE6891-AML: n=493; Beat-AML: n=477; blue and red violins, show Il1rl1 expression lower and higher than the median, respectively. Violin plots show median, the 25th and 75th percentiles, and whisker extend to the minimum and maximum. Wilcoxon rank-sum test was used.

Supplementary Fig. 3 Single cell RNA sequencing in BM from AML refractory and responder patients

A

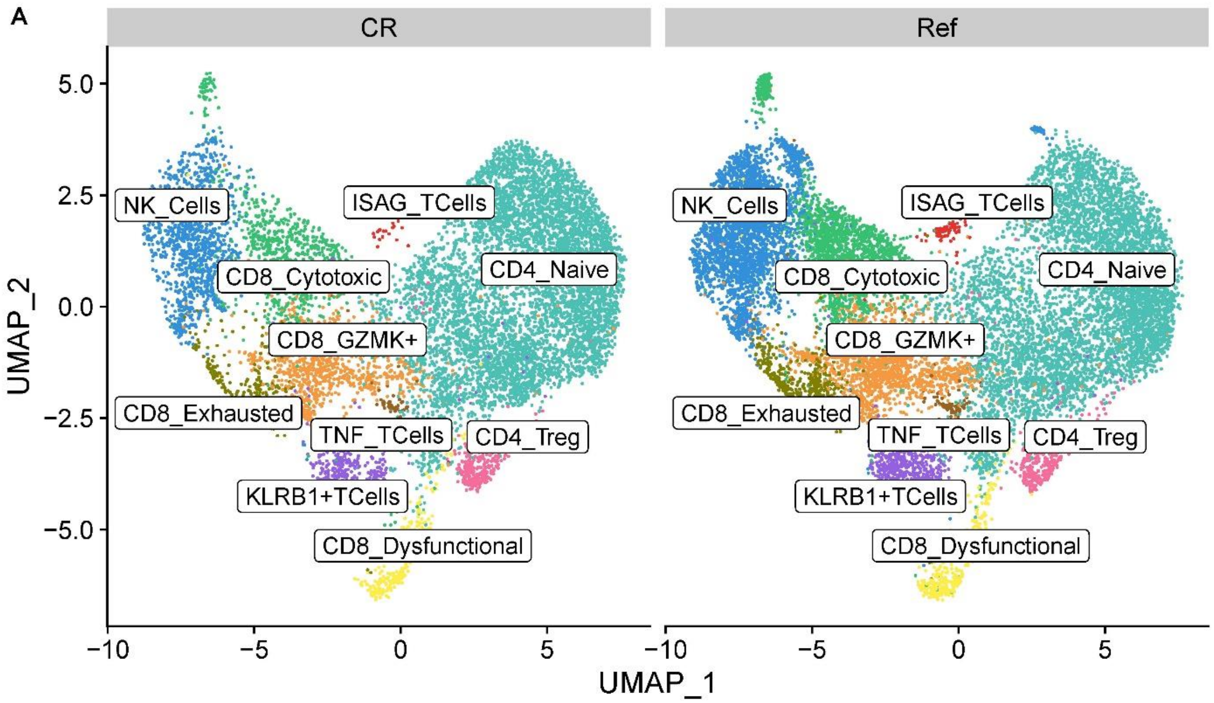

B

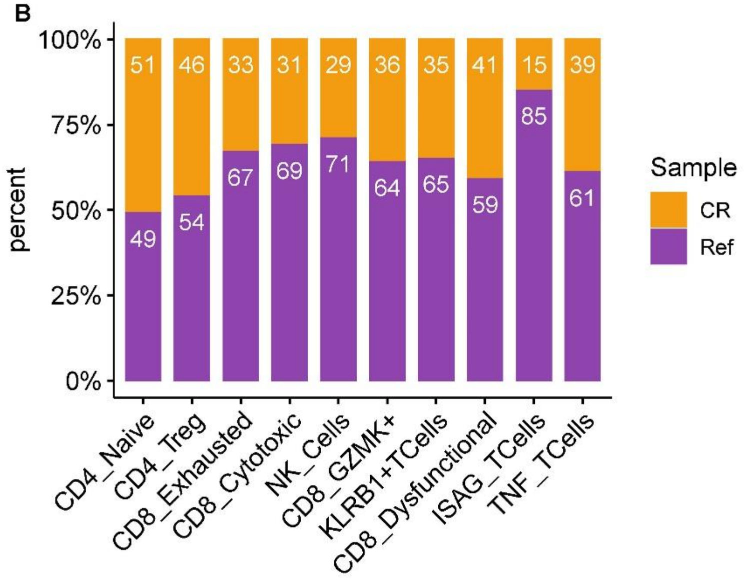

A, Uniform Manifold Approximation and Projection (UMAP) clustering of T cells and NK cells populations in AML patients' BM samples after chemotherapy induction comparing complete responders (CR, n=12) versus refractory patients (Ref., n=10).

B, Percentage of each T cell-cluster in CR versus Ref. samples.

Supplementary Fig. 4 Gating strategies for flow cytometric analysis

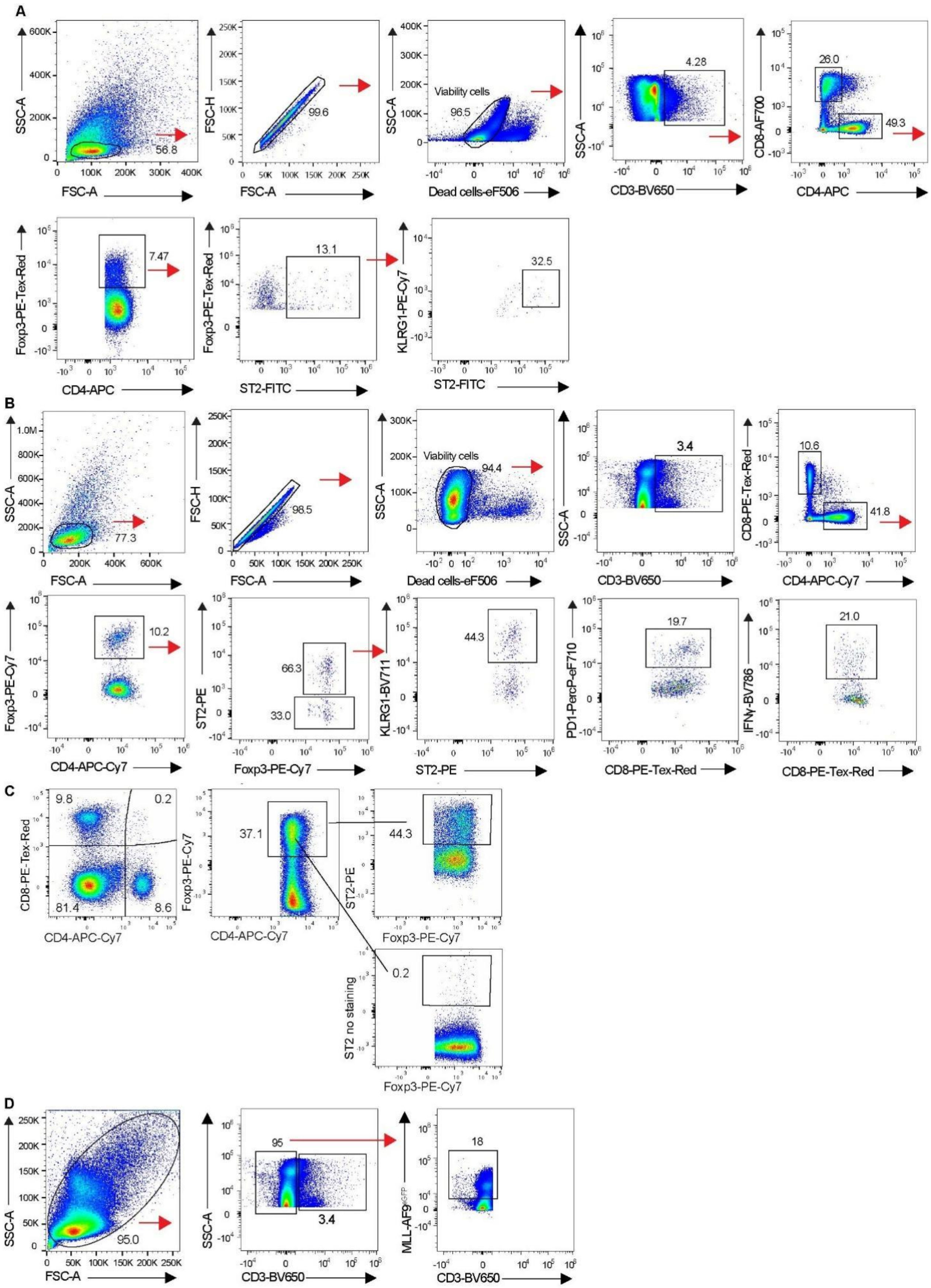

A, Gating strategy for KLRG1$^+$ST2$^+$ T_{reg} cells from AML patients' samples.
B, Gating strategy in murine AML samples for KLRG1$^+$ST2$^+$ T_{reg} cells (CD3$^+$T cells→CD4$^+$T cells→CD4$^+$Foxp3$^+$T cells → ST2$^+$Foxp3$^+$T cells → KLRG1$^+$ST2$^+$Foxp3$^+$T cells and for CD8$^+$PD-1$^+$ T cells and CD8$^+$IFNγ$^+$ T cells.
C, ST2 staining Fluorescence Minus One (FMO) control.
D, Gating strategy in murine AML samples for MLL-AF9^{eGFP+} cells (gated on CD3$^-$ cells).

Supplementary Fig. 5 ST2$^+$ T_{reg} cells and total T_{reg} cells in BM from patients with AML.

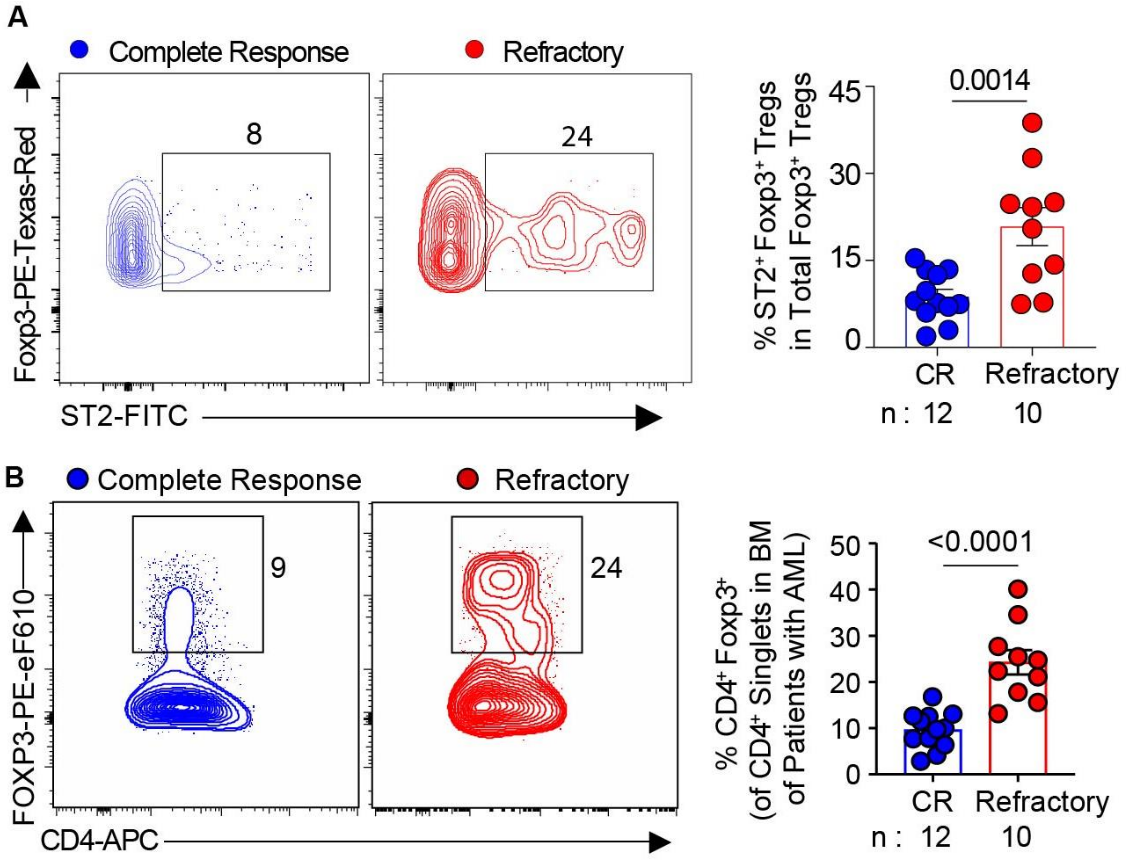

A, Representative flow data for AML patients' ST2+ T_{reg} cells and statistical quantities.
B, Representative flow data for AML patients' total Treg cells and statistical quantities.
Data are mean ± s.e.m. (error bar) and compared using two-sided Student's *t* test. Source data are provided as a Source Data file.

Supplementary Fig. 6 Frequencies of total ST2^{+} T_{reg} cells post MLL-AF9 or DNMT3A/FLT3^{ITD}-mutant leukemic cell challenge.

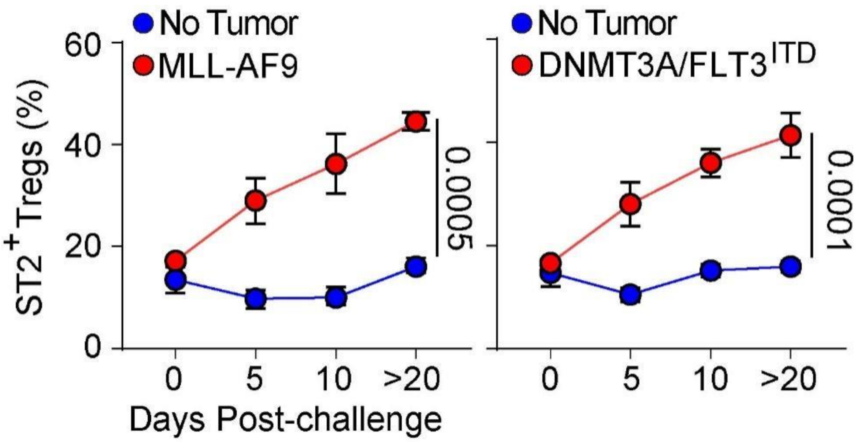

Frequencies of total ST2^{+} T_{reg} cells in the malignant BM niches on day 0, 5, 10, and >20 post MLL-AF9 or DNMT3A/FLT3^{ITD}-mutant leukemic cell challenge (10^5 per mouse). Data are mean ± s.e.m. (error bar) and compared using two-sided Student's *t* test. Source data are provided as a Source Data file.

Supplementary Fig. 7 Comparisons of percentages of T_{reg} cells positive for the indicated markers between paired $ST2^+$ T_{reg} cells and $ST2^-$ T_{reg} cells in the DNMT3A/FLT3^{ITD}-mutant model.

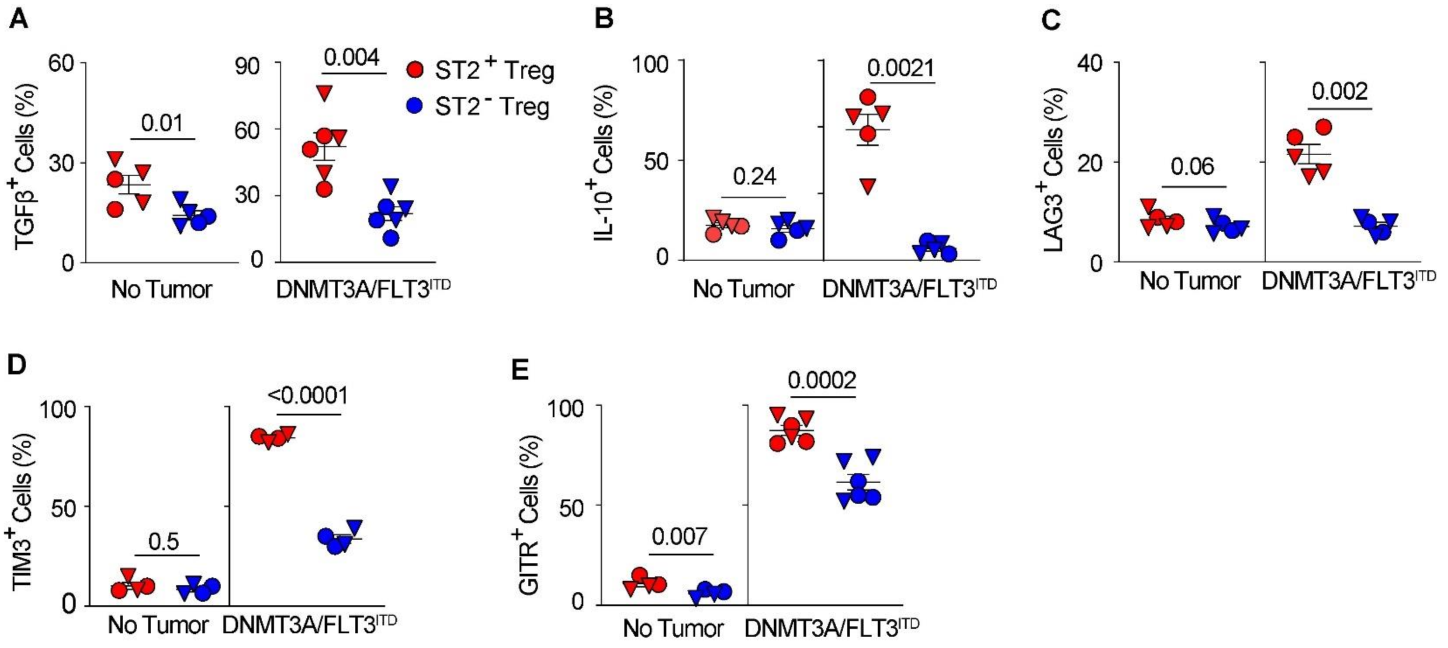

A-E, Cytokines TGFβ (n=5 for no tumor group, n=6 for tumor group) (**A**); IL-10 (n=5) (**B**); and T_{reg} cells functional drivers LAG3 (n=5) (**C**); TIM3 (n=5) (**D**) and GITR (n=4 for no tumor group, n=6 for tumor group) (**E**) post DNMT3A/FLT3^{ITD}-mutant leukemic cell challenge (10^5 per mouse) on day 10. Data are pooled from two independent replicates (shown as different shapes). The control group had no tumor cell transfer.
Data are mean ± s.e.m. (error bar) and compared using two-sided Student's *t* test. Source data are provided as a Source Data file.

Supplementary Fig. 8 Correlation analysis of indicated CD8 subsets with ST2^{+} T_{reg} cells in the DNMT3A/FLT3^{ITD}-mutant model.

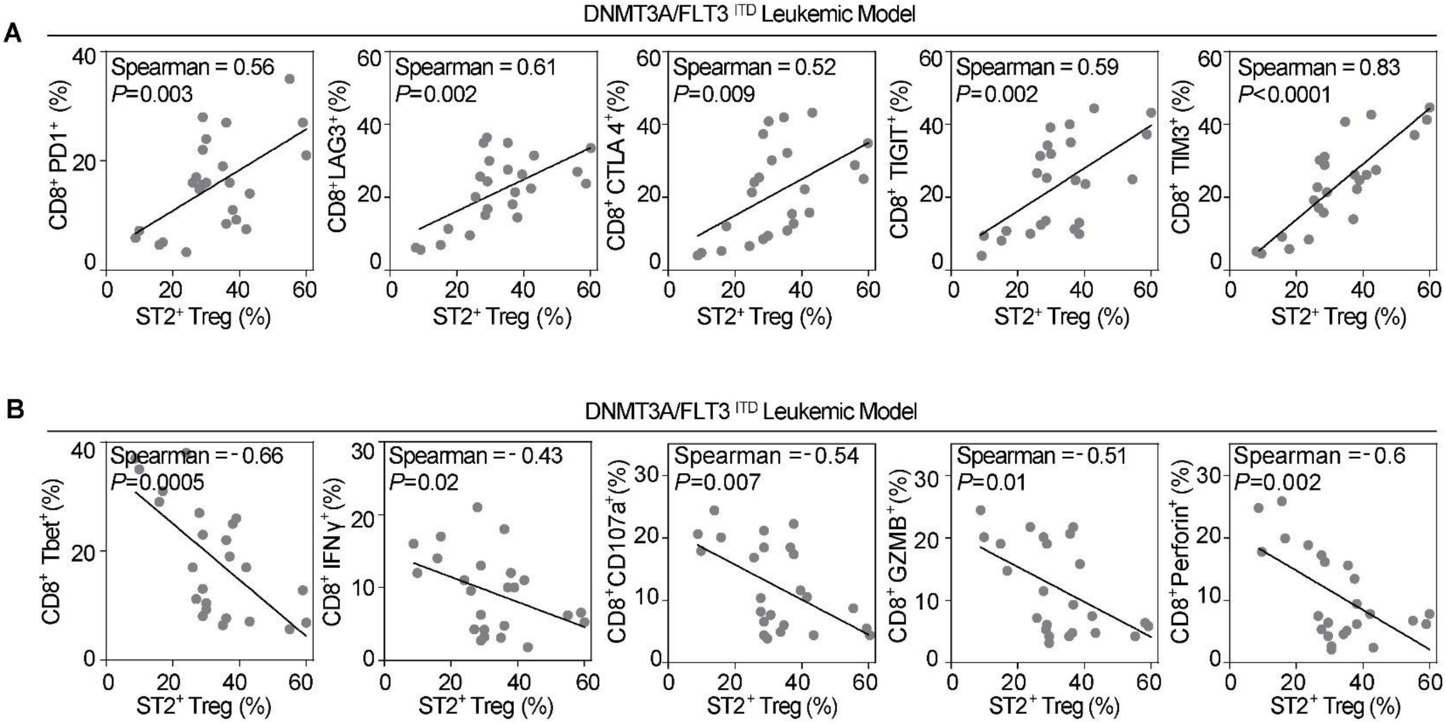

A-B, Correlation analysis of CD8^{+}PD1^{+}, CD8^{+}LAG3^{+}, CD8^{+}CTLA4^{+}, CD8^{+}TIGIT^{+} and CD8^{+}TIM3^{+} (**A**); and CD8^{+}Tbet^{+}, CD8^{+}IFNγ^{+}, CD8^{+}CD107a^{+}, CD8^{+}GZMB^{+} and CD8^{+}Perforin^{+} T cells (**B**) with ST2^{+} T_{reg} cells in the malignant BM niche post-DNMT3A/FLT3^{ITD} -mutant leukemic cell challenge (10^{5} per mouse, samples taken on day 5, day 10 and out of day 20 post leukemic challenge) (n=24). Spearman's correlation analysis was used.

Supplementary Fig. 9 Correlation analysis of CD4⁺ T effector cells and NK cells with ST2⁺ T_{reg} cells.

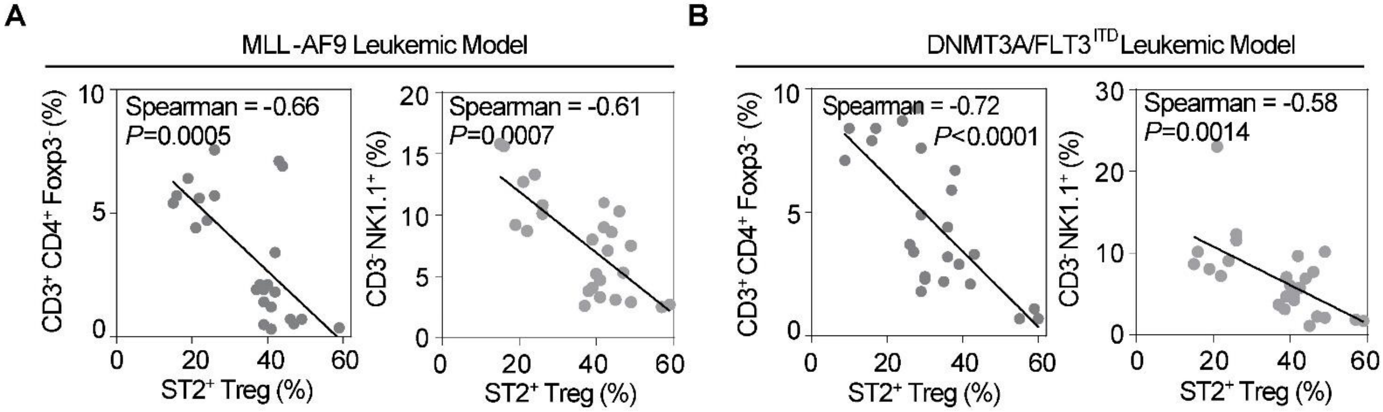

A-**B**, Correlation analysis of $CD4^+Foxp3^-$ T cells, and $CD3^-NK1.1^+$ NK cells with $ST2^+$ T_{reg} cells in the malignant BM niches post MLL-AF9 (n=27) **(A)** or DNMT3A/FLT3^{ITD}-mutant (n=24) **(B)** leukemic cell challenge (10^5 per mouse, samples taken between day 10 and day 21 post-AML), using Spearman's correlation analysis.

Supplementary Fig. 10 Statistically analyzed frequencies of total T_{reg} cells and activated T_{reg} cells in the MLL-AF9 leukemic model adoptive transfer receiving $ST2^{-/-}Foxp3^{eGFP+}$ T_{reg} cells versus WT $Foxp3^{eGFP+}$ T_{reg} cells.

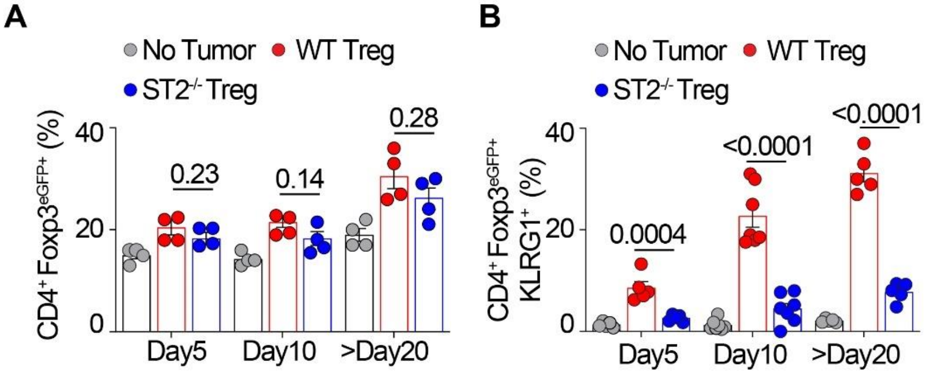

A-B, Total T_{reg} cells (gated on $CD3^+$ population, $CD4^+eGFP^+$, Foxp3 tagged by eGFP) (n=4) (**A**); and activated T_{reg} cells ($CD4^+KLRG1^+eGFP^+$) (n=5 on day5 and day>20, n=7 on day10) (**B**) in the malignant BM niches on the indicated days post MLL-AF9 leukemic cell challenge (10^5 per mouse).

Data are mean ± s.e.m. (error bar) and compared using two-sided Student's *t* test. Source data are provided as a Source Data file.

Supplementary Fig. 11 Frequencies of MLL-AF9^{eGFP} cells in the malignant BM niches on day 10 post 10^6 leukemic cell challenge and adoptive transfer of ST2$^{-/-}$Foxp3^{eGFP+} T_{reg} cells versus WT Foxp3^{eGFP+} T_{reg} cells.

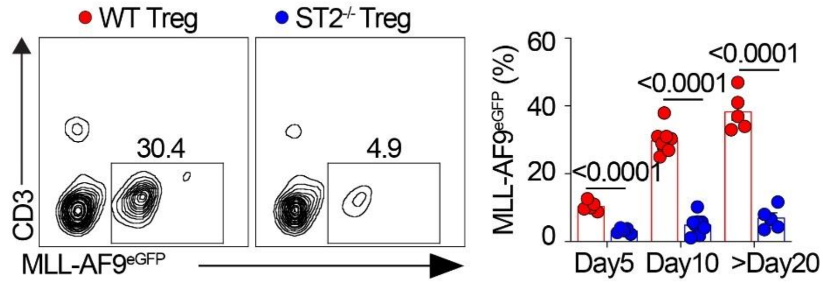

Representative flow plots gated on CD3$^-$ population, and statistical analysis on the indicated days (n=5 on day5 and day>20, n=7 on day10). Data are mean ± s.e.m. (error bar) and compared using two-sided Student's *t* test. Source data are provided as a Source Data file.

Supplementary Fig. 12 Number of CD8 T cells and frequencies of proliferating CD8 T cells post MLL-AF9^{eGFP} leukemic cell challenge.

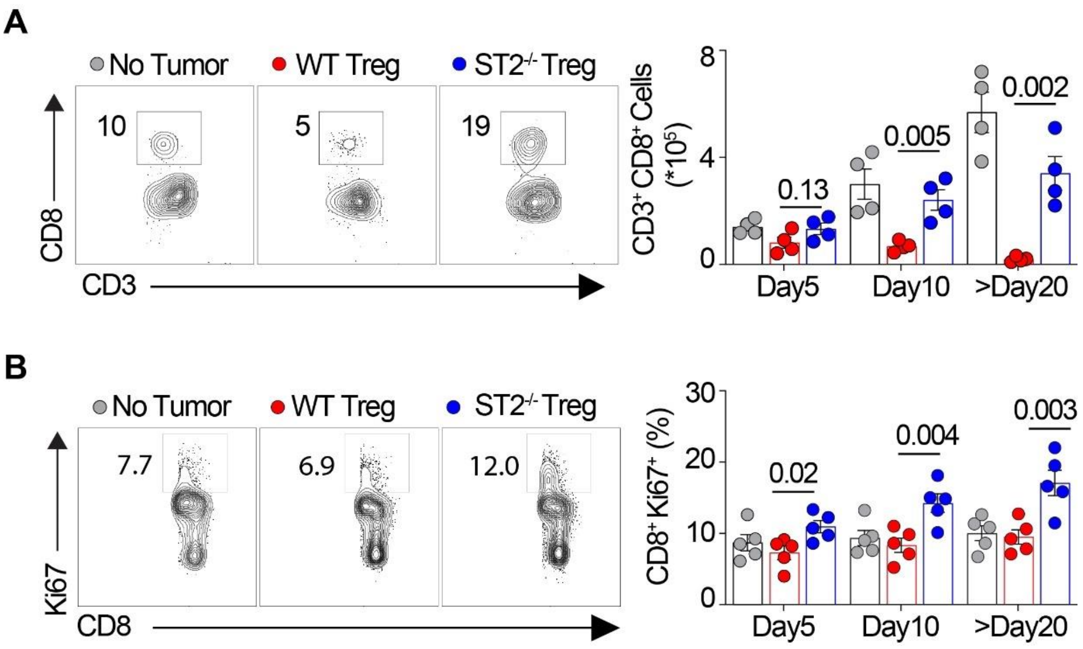

A-**B**, Representative flow plots and counts of $CD3^+CD8^+$ T cells (gated on $CD3^+$ T cells) (n=4) (**A**); and frequencies of $CD8^+Ki67^+$ T cells (gated on $CD3^+CD8^+$T cells) (n=5) (**B**) in the malignant BM niches on the indicated days. Data are mean ± s.e.m. (error bar) and compared using two-sided Student's *t* test. Source data are provided as a Source Data file.

Supplementary Fig. 13 WT-1 expression on murine MLL-AF9.

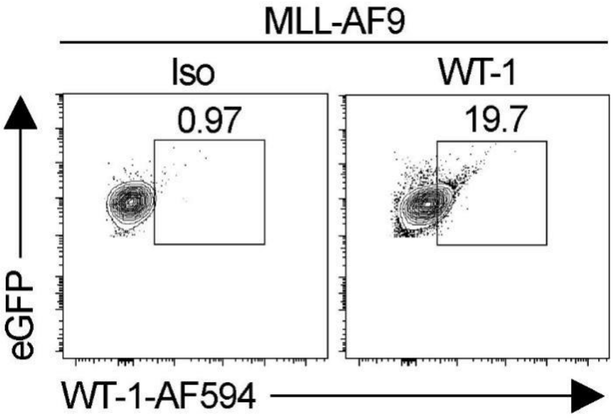

Representative flow plots of WT1 expression on sorted C57BL/6 MLL-AF9^{eGFP} leukemic cells.

Supplementary Fig. 14 NanoString analysis of naive T_{reg} cells sorted from $Tbet^{-/-}$ and WT mice and frequencies of $ST2^+$ and total T_{reg} cells gated in T_{reg} cells sorted from Tbet-/- and WT mice.

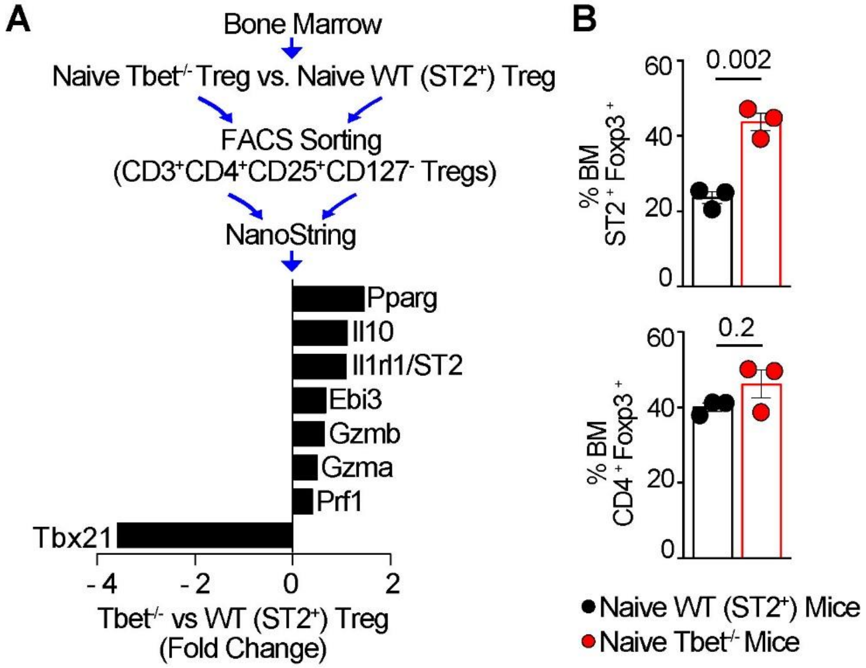

A, NanoString analysis of naive T_{reg} cells sorted from $Tbet^{-/-}$ and WT mice, increased transcripts of key molecules needed for T_{reg} cell activation, tissue repair, and pro-cytotoxicity function. Data are shown as ratio of log2-transformed fold change.

B, Frequencies of $ST2^+$ and total T_{reg} cells gated on $CD3^+CD4^+$ in the naive T_{reg} cells sorted from $Tbet^{-/-}$ and WT mice (n=3). Data are mean ± s.e.m. (error bar) and compared using two-sided Student's *t* test. Source data are provided as a Source Data file.

Supplementary Fig. 15 Functional phenotypes of BM-derived ST2$^+$ TME (WT) T_{reg} cells distinguishing among ST2-expressing versus ST2-negative cells.

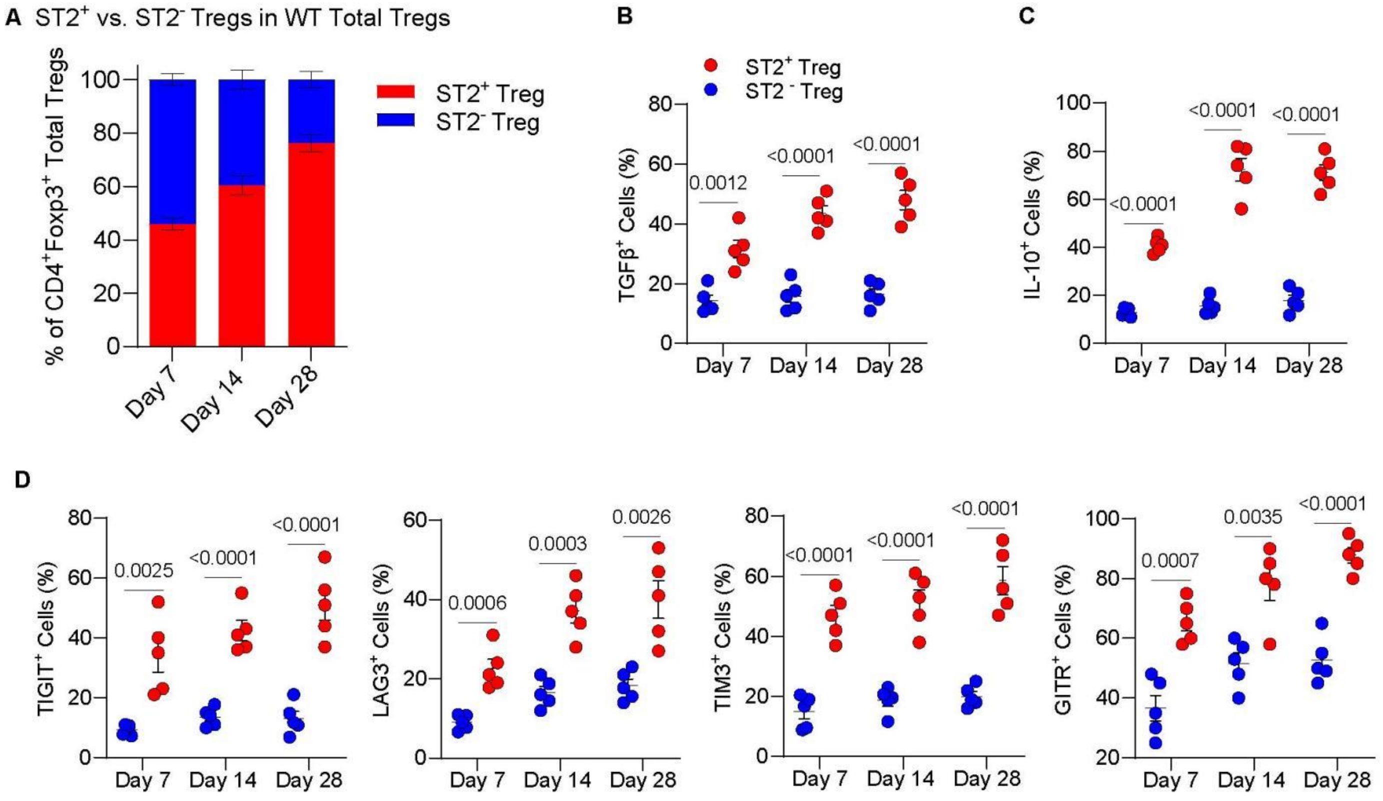

A-**D**, Frequencies of ST2$^+$ versus ST2$^-$ T_{reg} cells in WT BM total T_{reg} cells at Days 7, 17, 28 post-MLL-AF9 leukemic cells transfer in the adoptive transfer model with 20% WT T_{reg} cells (**A**); Frequencies of TGFβ expressing cells in ST2$^+$ versus ST2$^-$ T_{reg} cells (**B**); Frequencies of IL-10 expressing cells in ST2$^+$ versus ST2$^-$ T_{reg} cells (**C**), Frequencies of cells expressing co-inhibitory receptors (TIGIT, LAG3, TIM3, and GITR) in ST2$^+$ versus ST2$^-$ T_{reg} cells (n=5) (**D**). WT BM total T_{reg} cells was gated on eGFP$^+$CD4$^+$ T cells. Data are mean ± s.e.m. (error bar) and compared using two-sided Student's *t* test. Source data are provided as a Source Data file.

Supplementary Fig. 16 Statistical analysis of different CD8 subsets in mice with or without MLL-AF9 leukemia transferred with 20% WT or ST2$^{-/-}$ or Tbet$^{-/-}$ T_{reg} cells.

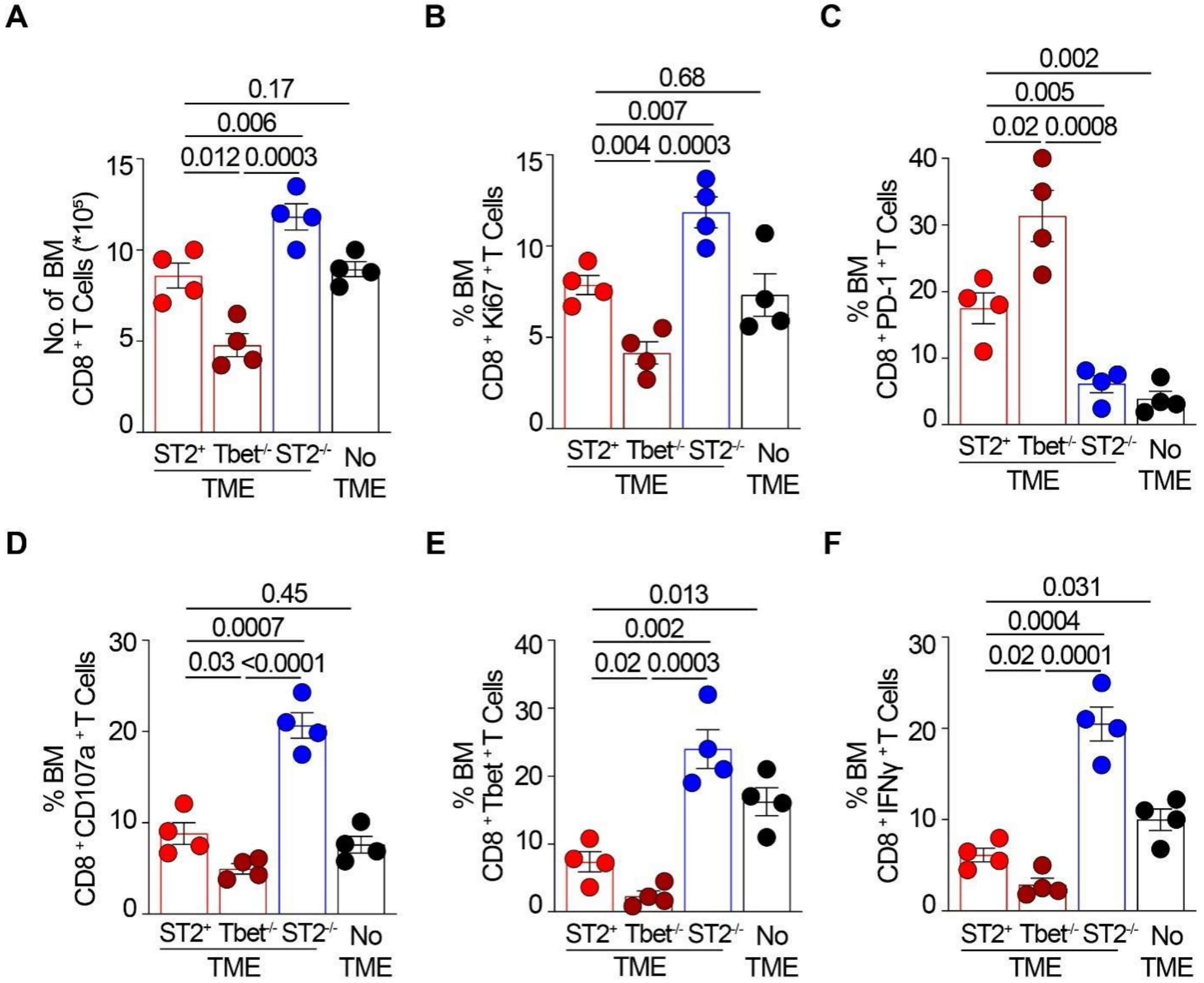

A-**F**, Number of BM CD8 T cells (**A**) and frequencies of BM CD8$^+$Ki67$^+$ T cells (**B**), CD8$^+$PD-1$^+$ T cells (**C**), CD8$^+$CD107a$^+$ T cells (**D**), CD8$^+$Tbet$^+$ T cells (**E**) and CD8$^+$IFNγ$^+$T cells (**F**) among the ST2$^{-/-}$ T_{reg} cells versus WT (ST2$^+$) T_{reg} cells versus Tbet$^{-/-}$ T_{reg} cells versus no tumor transfer group on day 14 post leukemic cell challenge. CD8 T cells were gated on GFP$^-$CD3$^+$ T cells (n=4). Data are mean ± s.e.m. (error bar) and compared using two-sided Student's *t* test. Source data are provided as a Source Data file.

Supplementary Fig. 17 Statistically analyzed direct killing of CD4⁺ T effector cells and NK cells by T_{reg} cells via flow imaging.

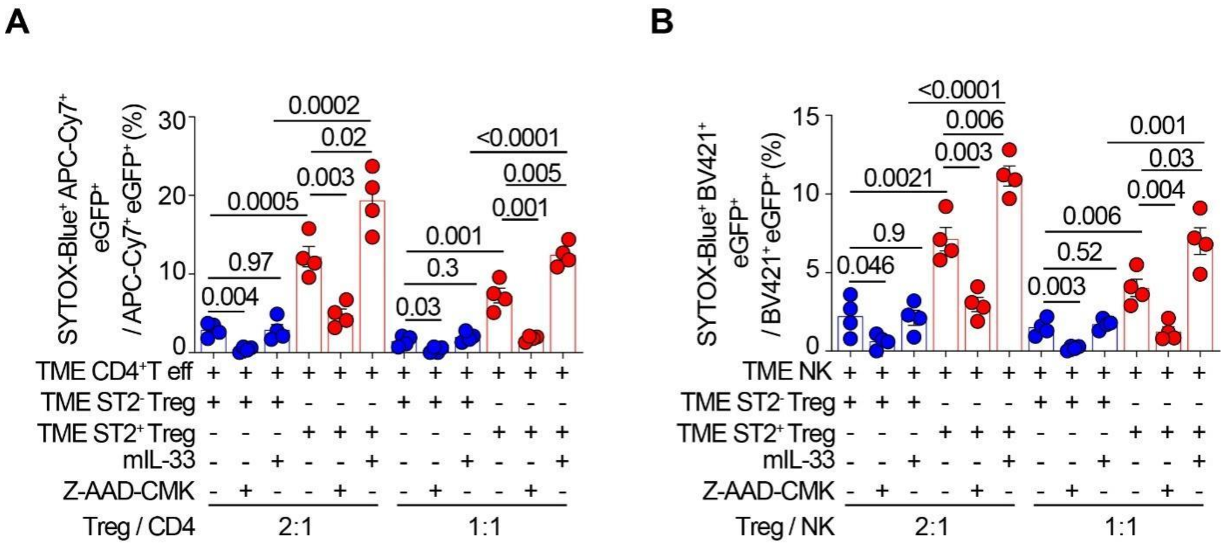

A-**B**, Percentages of lysed TME-derived CD4⁺ T effector cells (**A**) and NK cells (**B**) in each coculture condition measured by flow imaging and SYTOX release (n=4). Data are mean ± s.e.m. (error bar) and compared using two-sided Student's *t* test. Source data are provided as a Source Data file.

Supplementary Fig. 18 HPLC, SPR and pharmacokinetics study of IgG-281 and IgG-282.

A

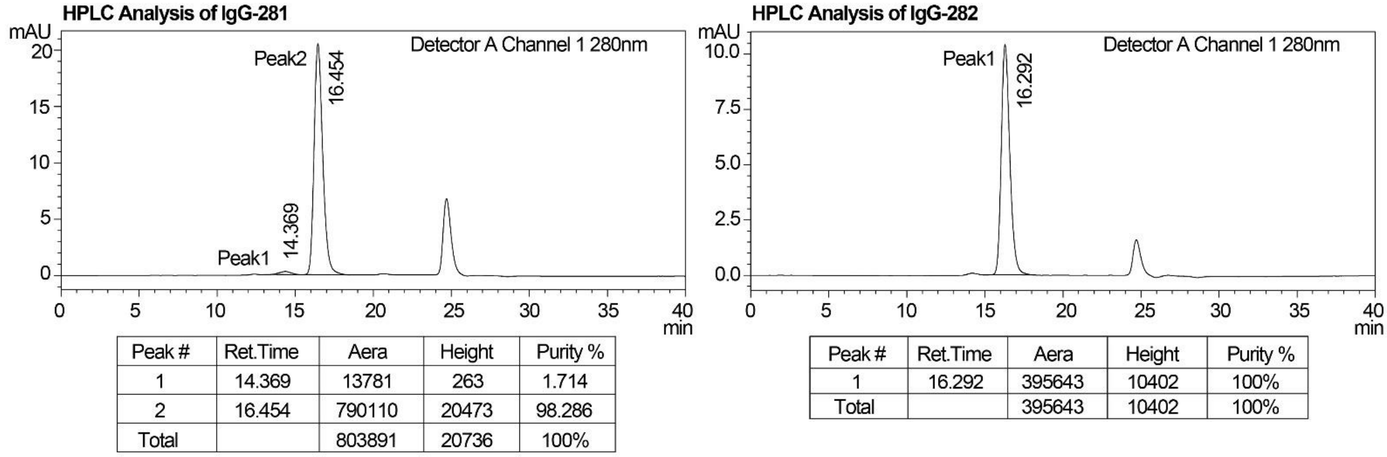

Peak #	Ret.Time	Aera	Height	Purity %
1	14.369	13781	263	1.714
2	16.454	790110	20473	98.286
Total		803891	20736	100%

Peak #	Ret.Time	Aera	Height	Purity %
1	16.292	395643	10402	100%
Total		395643	10402	100%

B

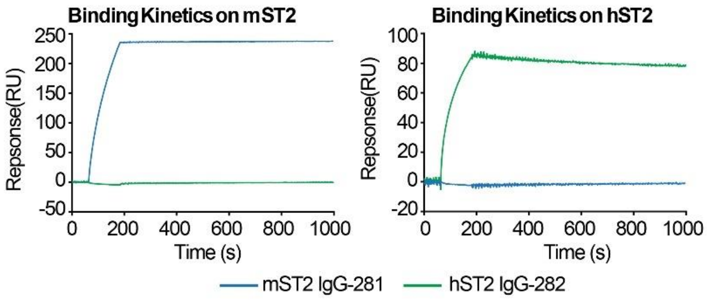

Antibody	Ka (1/Ms)	Kd (1/s)	KD (M)	t 1/2 (s)
IgG-281	6.53E+04	2.48E-08	3.81E-13	27897110.2
IgG-282	1.38E+05	7.86E-05	5.69E-10	8819.7

C

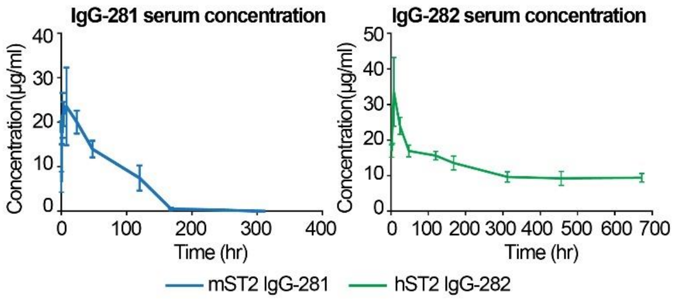

Antibody	Half-life	Half-life	T max	C max	C last	AUC all
IgG-281	70 hrs	2.9 days	8 hrs	24 μg/ml	0.5 μg/ml	1883
IgG-282	424 hrs	17.7 days	8 hrs	33 μg/ml	9.4 μg/ml	8055

A, HPLC of IgG-281 and IgG-282. Tables show the purity of the two antibodies.
B, Surface plasmon resonance (SPR) analysis and indexes of the interaction between ST2 antibodies and surface ligands including the association constant (K_a), dissociation constant (K_d), equilibrium constant (K_D), and binding half-life ($t_{1/2}$).
C, Pharmacokinetics study of IgG- 281 and IgG-282 in vivo employing mice of the same background used in Fig. 7 (n=5). Table shows half-lives, the time point with the highest concentration (T_{max}), the maximum concentration (C_{max}), concentration at the last timepoint (C_{last}), and the area under the curve for the study (AUC_{all}, hr*μg/mL). Data are mean ± s.e.m.

Supplementary Fig. 19 *In vitro* apoptosis and proliferation of TME-derived CD8 T cells treated with increasing IgG-281 concentrations.

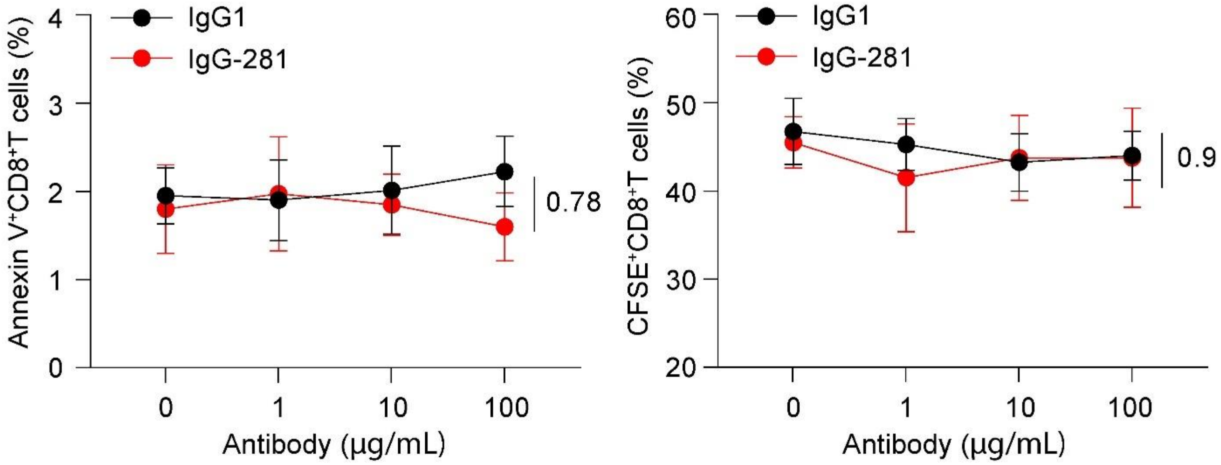

Data are mean ± s.e.m. (error bar) and compared using two-sided Student's *t* test (n=3). Source data are provided as a Source Data file.

Supplementary Fig. 20 Frequencies of immune cells parameters in the malignant BM niches on day 21 after the first administration of IgG1 control versus IgG-281 and anti-PD-1.

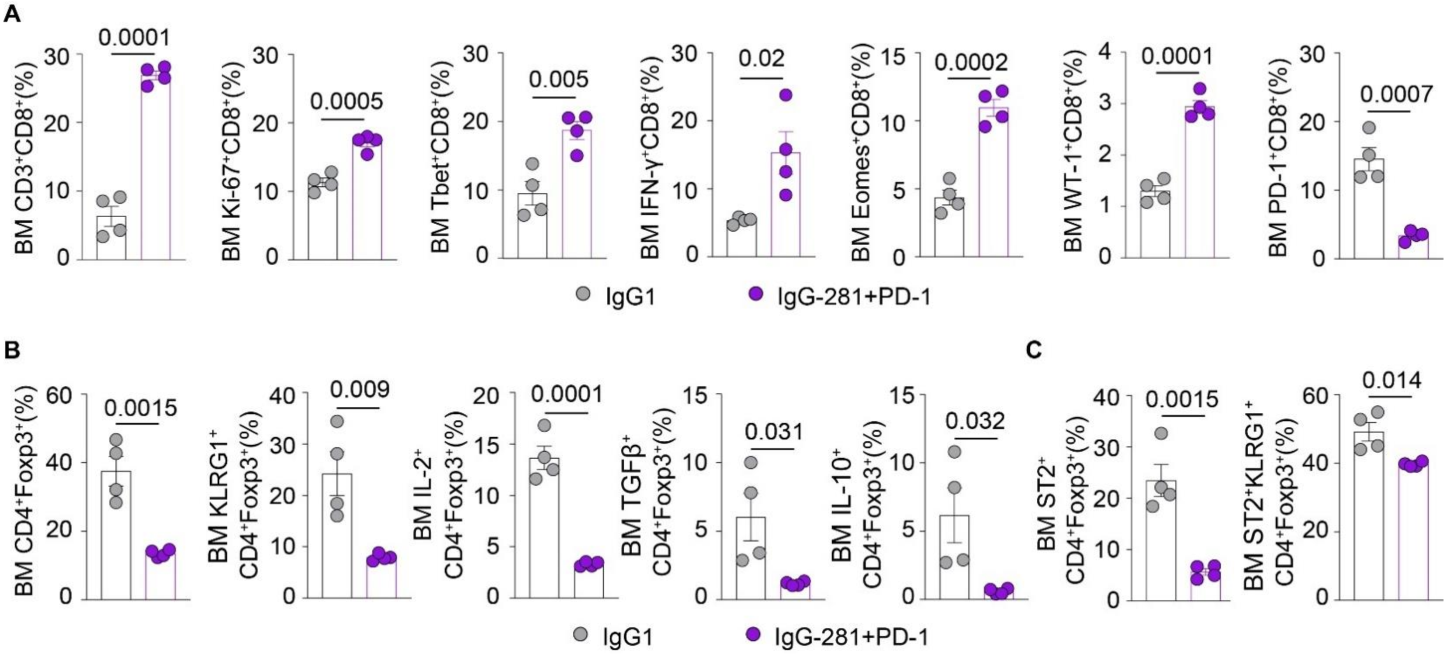

A, Statistically analyzed frequencies of $CD3^+CD8^+$ and $Ki67^+$, $Tbet^+$, $IFN\gamma^+$, Eomes+, $WT\text{-}1^+$, and $PD\text{-}1^+$ T cells in the BM niche of mice treated with IgG1 versus IgG-281+anti-PD-1 on Day 21 post the first administration of the antibodies (n=4).

B, Statistically analyzed frequencies of $CD4^+$ and $Foxp3^+$, $KLRG1^+Foxp3^+$, $IL\text{-}2^+Foxp3^+$, $TGF\beta^+Foxp3^+$, $IL\text{-}10^+Foxp3^+$T cells in the BM niche of mice treated with IgG1versus IgG-281+ anti-PD-1 on Day 21 after the first administration of the antibodies (n=4).

C, Statistically analyzed frequencies of $CD4^+Foxp3^+ST2^+$ and $CD4^+Foxp3^+$ $ST2^+KLRG1^+$ T_{reg} cells in the BM niche of mice treated with IgG1 vs IgG-281+anti-PD-1 on Day 21 post the first administration of the antibodies (n=4).

Data are mean ± s.e.m. (error bar) and compared using two-sided Student's *t* test. Source data are provided as a Source Data file.

Supplementary Fig. 21 Anti-ST2 antibody promotes abatement of ST2⁺T$_{reg}$ cells to extend survival in HLA-A2-matched humanized AML model.

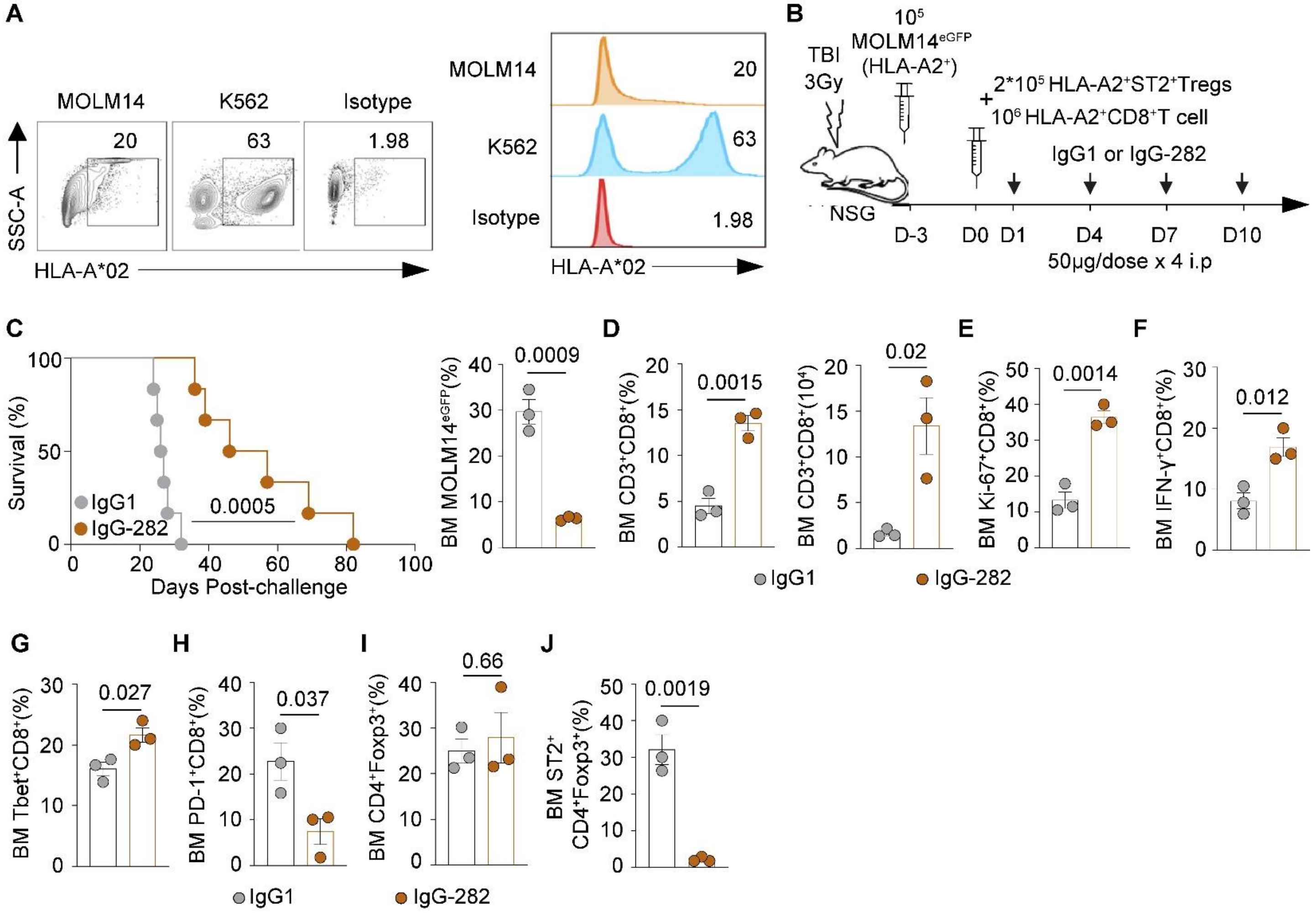

A, HLA-A2 expression on MOLM14 cells gated on total cells.
B, Experimental scheme of administration IgG1 control or IgG-282 to HLA-A2-matched humanized NSG leukemic model.
C, Survival analysis and the frequency of MOLM14 cells in leukemic cell-bearing NSG mice treated with IgG1 control or IgG-282.
D, Frequencies and absolute number of CD3⁺CD8⁺T cells in the malignant BM niches on day 21 after the first administration of IgG1 control versus IgG-282.
E-**H**, Statistically analyzed frequencies of Ki67⁺CD8⁺ (**E**), IFNγ⁺CD8⁺ (**F**), Tbet⁺CD8⁺ (**G**), PD-1⁺CD8⁺ T cells (**H**) in the BM niche of mice treated with IgG1 vs IgG-282 at Day 21 post leukemia challenge.
I-J Statistically analyzed frequencies of CD4⁺Foxp3⁺ (**I**) and ST2⁺CD4⁺Foxp3⁺ T$_{reg}$ cells (**J**) in the BM niche of mice treated with IgG1 vs IgG-282 at Day 21 post leukemia challenge.
Data represents two independent replicates (n=3). Data are mean ± s.e.m. (error bar) and compared using two-sided Student's *t* test. Source data are provided as a Source Data file.

Supplementary Table 1. Demographics of patients with AML

Age	Gender	Race	Cyto ISCN	FLT3	NPM1	Treatment	% blasts post-induction	Response
60	Female	Caucasian	46,XX[20]	Pos	Pos	Tosedostat + DAC	15.50	Refractory
46	Male	Caucasian	43,XY,del(1)(q32),der(1)del(1)(p36.1)t(1;7)(q21;q11.2)add(7)(q36), der(2)inv(2)(p23q35)add(2)(p11.2), del(3)(q21q2?1),del(4)(q22q25),add(5)(q31),-7, der(7)t(1;7),add(8)(p11.2),-11,-12,-13,add(14)(p11.2),add(15)(p11.2), del(15)(q?11.2q24),-16,del(17)(p11.2),add(20)(p13),add(21)(q22),+2~4mar[cp6]/ 41~43,sl,-18[cp3]/41~43,sl,del(18)(p11.1p11.2)[cp3]//46,XX[8]	Neg	Neg	IAP	24.00	Refractory
40	Female	Caucasian	46,XX[20]	Pos	Pos	7 + 3 + Sorafenib	0.50	Refractory
57	Female	Caucasian	ND	ND	ND	FLAM	0.00	CR
56	Female	American Indian or Alaska Native	46XX[20]	ND	ND	IAP	2.00	Refractory
26	Male	Caucasian	46,XY[20]	Neg	Neg	7 + 3 + GO	0.00	CR
68	Male	Unknown	46, XY[20]	Neg	Neg	7 + 3 (DNR90)	0.00	CR
24	Female	Hispanic	46,XX,inv(16)(p13q22)(17]/47,sdl,+22[3]	Neg	Neg	7 + 3 (DNR90)	0.00	CR
63	Female	Caucasian	46,XX[20]	Neg	ND	Benda + Idarubicin	5.00	Refractory
29	Female	Caucasian	46,XX,[20]	Neg	Neg	GCLAC	0.00	CR
69	Female	Caucasian	45,X,-X,del(20)(q12)[4]/43,sl,add(3)(q11.2),der(4)t(3;4)(q21;q21),-5,-7,-15,-16,+r,+mar1[7]/44,sdl,+mar2[3]/38,sl,add(3)(q11.2),der(4)t(3;4)(q21;q21),-5,-7,-12,dic(15;21)(p13;p13),-16,-18,-21,add(22)(p11.2)[4]	Neg	Neg	Benda + Idarubicin	24	Refractory
64	Male	Pacific Islander	48,XY,add(3)(q11.2),add(5)(q22),+10,+11,add(12)(q15),+13,-16,-17,+22[5]/49,sl,+13[5]/48,sl,add(17)(p11.2),del(20)(q11.2)[1]/46,XY[9].	Neg	ND	GCLAC	12.00	Refractory
39	Male	Unknown	46,XY,t(8;21)(q22;q22)[8]/45,sl,-7[11]/46,XY[1]	Neg	Neg	GCLAC	0.00	CR
51	Male	Caucasian	46,XY[20]	Neg	Neg	GCLAC	0.00	CR
62	Female	Caucasian	46,XX[20]	Neg	Neg	GCLAC + Cord	5.00	Refractory
65	Male	Caucasian	48,XY,+X,+21[20]	Neg	Neg	Tosedostat + ARAC	14.25	Refractory
82	Male	Caucasian	46,XY, t(5;12) with rearrangements involving 12q13	Neg	Neg	Benda + IDA	20.00	Refractory
36	Male	Pacific Islander	46,XY[20]	Neg	Neg	7 + 3 (DNR90)	0.00	CR
63	Female	American Indian or Alaska Native	46,XX [20]	Neg	Neg	7 + 3 (DNR60)	0.00	CR
53	Male	Caucasian	46,XY	Neg	Neg	IAP	0.00	CR
46	Female	unknown	46,XX[20]	Pos	Neg	7+3 (IDA)	2	CR

Supplementary Table 2. Nanostring selected transcripts of BM-derived WT T_{reg} cells and Tbet$^{-/-}$ T_{reg} cells sorted from sex- and age-matched normal naïve WT and Tbet$^{-/-}$ mice.

Gene Name	BM Treg: Tbet KO naive	BM Treg: WT naive	KO/WT
Pparg	26.75	9.64	2.77
Il10	49.04	20.65	2.37
Il1rl1	178.33	81.22	2.19
Ebi3	47.56	33.04	1.44
Gzmb	13.38	15.14	0.88
Gzma	7.43	6.88	1.08
Prf1	8.92	8.26	1.08
Tbx21	1.49	17.9	0.08

Supplementary Table 3. Selected genes represented in the heatmap of Treg cells sorted from no leukemia cells transferred and malignant BM of mice in which ST2$^{-/-}$ Treg cells versus WT (ST2^{+}) Treg cells versus Tbet$^{-/-}$ Treg cells transferred.

Gene symbol	WT No tumor-1	WT No tumor-2	WT No tumor-3	Tumor ST2-/-Treg-1	Tumor ST2-/-Treg-2	Tumor ST2-/-Treg-3	Tumor WT Treg-1	Tumor WT Treg-2	Tumor WT Treg-3	Tumor Tbet-/-Treg-1	Tumor Tbet-/-Treg-2	TumorTbet-/-Treg-3
Foxp1	6.60	6.50	6.50	6.20	6.20	6.20	5.20	5.30	5.30	4.70	4.70	4.70
Sox4	5.79	5.82	5.88	5.33	5.39	5.29	3.95	3.90	3.82	2.07	2.23	2.32
Tcf3	7.26	7.32	7.39	7.06	6.94	6.98	5.79	5.74	5.72	4.68	4.61	4.62
Bach2	7.64	7.64	7.65	7.31	7.27	7.29	5.73	5.73	5.77	2.50	2.65	2.50
Tnfrsf19	4.12	4.30	4.27	3.81	3.98	3.78	2.78	2.74	2.66	-0.83	-1.08	-0.69
Lgr5	4.97	4.87	4.92	4.47	4.35	4.51	3.44	3.54	3.54	0.19	-0.01	-0.15
Id3	4.03	4.12	4.14	3.60	3.79	3.39	2.48	2.49	2.12	-0.33	0.64	-0.31
Lef1	6.19	6.21	6.16	5.61	5.54	5.44	5.21	5.19	5.16	4.16	4.13	4.03
Gzma	3.84	3.46	3.71	2.44	2.94	3.02	4.34	4.46	4.26	5.34	5.47	5.32
Il1rl1	3.44	3.48	3.40	3.71	3.61	3.67	4.12	4.16	4.26	5.18	5.13	5.21
Il10	1.36	1.43	1.86	1.17	1.22	1.82	2.47	2.43	2.85	3.65	3.54	3.72
il1r2	3.58	4.01	3.98	3.57	3.44	3.76	4.34	4.57	4.43	4.98	5.17	4.94
il9r	1.76	1.72	1.53	0.56	0.87	1.20	1.35	1.24	1.21	3.63	3.61	3.62
il6	-0.11	-0.06	-0.87	-1.93	-3.04	-3.04	-0.69	-1.48	-0.42	1.81	2.01	1.64
Pdcd1	1.90	2.23	2.67	2.72	3.40	3.24	3.40	3.20	3.41	5.03	4.93	5.03
Gata3	4.13	4.13	4.26	4.89	4.87	4.99	5.30	5.21	5.32	6.50	6.42	6.45
Lag3	1.77	1.59	1.79	2.18	2.21	2.25	2.91	3.02	2.89	4.13	4.15	4.41
Il13	-2.55	-1.47	-1.87	-2.08	-0.85	0.12	-0.96	-0.89	-0.70	6.03	5.94	5.91
Cd69	4.10	4.10	4.30	4.30	4.30	4.20	4.20	4.30	4.40	4.90	4.90	5.10
Gzmb	4.66	4.55	4.62	5.14	5.08	4.99	6.49	6.54	6.54	6.50	6.42	6.44
Gzmk	1.01	0.83	1.00	1.56	0.41	1.99	3.97	3.94	3.70	4.00	3.89	4.50
Areg	-1.50	-0.82	-2.60	-2.08	-0.69	-1.07	-0.19	-0.51	0.50	2.01	2.02	1.59
Pparg	1.90	1.99	1.67	2.07	2.91	2.74	3.77	3.63	3.58	4.13	4.08	4.31
Ebi3	3.88	4.38	4.01	4.33	4.26	4.37	4.80	4.77	4.71	5.15	5.16	5.24
Prf1	0.91	1.23	1.06	1.67	1.68	1.74	2.98	2.82	2.87	3.77	3.61	3.62
Eomes	0.91	1.23	1.06	1.67	1.68	1.74	2.98	2.82	2.87	3.77	3.61	3.62

Supplementary Table 4. Antibodies used for the flow cytometry analyses Mouse

Antibody	Company	Clone	Fluorochrome	Dilution
CD45	eBioscience	30-F11	PE/eF506	1:100
CD45.1	eBioscience	A20	PE/APC	1:100
CD3e	eBioscience	145-2C11	PE-Cy5/BV650	1:100
CD90	eBioscience	30-H12	FITC/ PE-Cy7	1:100
CD4	eBioscience	GK1.5	PercP-eFluor710	1:100
CD8α	eBioscience	53-6.7	BV605/650/785	1:100
IFNγ	eBioscience	XMG1.2	PB450/PerCP-Cy5.5	1:50
Perforin	eBioscience	eBioOMAK-D	APC/FITC	1:50
Granzyme B	eBioscience	NGZB	APC/BV421	1:50
Granzyme A	eBioscience	CB9/GzA-3G8.5	BV421/APC	1:50
Foxp3	eBioscience	FJK-16s	PE-Cy7/BV421	1:50
KLRG1	eBioscience	2F1	PE-Cy7/APC	1:50
CD44	eBioscience	IM7	APC/BV421	1:50
CD62L	eBioscience	MEL-14	PE-Cy5/APC	1:50
ST2	mdbioproduct	Dj8	PE/FITC	1:50
NK1.1	eBioscience	PK136	eF780	1:50
Tbet	eBioscience	TB10	eF660	1:50
TGFβ	eBioscience	TW7-16B4	PE-Cy7	1:50
TNFα	eBioscience	MP6-XT22	APC	1:50
IL-10	eBioscience	JES5-16E3	AF700	1:50
LAG3	eBioscience	3DS223H	APC/APC-eF780	1:50
TIM3	eBioscience	F38-2E2	APC/SB780	1:50
TIGIT	eBioscience	MBSA43	PercP-eFluor710/AF594	1:50
PD-1	eBioscience	eBioJ105 (J105)	PE-eFluo610	1:50
GITR	eBioscience	eBioAITR	APC/PE-Cy7	1:50
Ki67	eBioscience	SolA15	eF660	1:50
CD107a	eBioscience	1D4B	PE/eF660	1:50
CTLA4	eBioscience	UC10-4B9	PercP-eFluor710/APC	1:50
CD11b	eBioscience	M1/70	PE-Cy7/APC	1:50
Ly6C	eBioscience	HK1.4	eFluor450	1:50
Ly6G	eBioscience	1A8-Ly6g	PE/APC	1:50
F4/80	eBioscience	BM8	APC/FITC	1:50
B220	eBioscience	RA3-6B2	SB600	1:50
NFIL3	eBioscience	S2M-E19	AF647	1:50
GATA3	eBioscience	TWAJ	PE-eFluor610/AF700	1:50
BATF	eBioscience	MBM7C7	PE	1:50
Bcl6	eBioscience	BCL-DWN	APC	1:50
Blimp-1	eBioscience	5E7	AF488	1:50
CCR1	Biolegend	S15040E	APC	1:50
CCR2	Biolegend	SA203G11	FITC	1:50
CCR5	Biolegend	HM-CCR5	APC	1:50
CCR6	Biolegend	29-2L17	APC	1:50
CCR7	Biolegend	4B12	PE-Cy7	1:50
CCR8	Biolegend	SA214G2	FITC	1:50
CCR9	Biolegend	9B1	FITC	1:50
CXCR3	Biolegend	CXCR3-173	APC	1:50
CXCR4	Biolegend	L276F12	APC-Cy7	1:50
CXCR6	Biolegend	SA051D1	APC-Cy7	1:50

Human

CD4	eBioscience	OKT4	PE/PerCP-eFluor710	1:50
CD8	eBioscience	OKT8	FITC/APC	1:50
IFNγ	eBioscience	4S.B3	PE/APC	1:20
Granzyme B	eBioscience	GB11	PE	1:20
ST2	mdbioproduct	B4E6	FITC	1:20
Tbet	Biolegend	4B10	PE-Cy7	1:20
Foxp3	Biolegend	QA18A03	PE	1:10
Ki67	eBioscience	SolA15	eFluor 660	1:20
CD107a	eBioscience	eBioH4A3	PE	1:20
PD-1	eBioscience	MIH4	PE	1:20
KLRG1	eBioscience	13F12F2	PerCP-eFluor710	1:20

Supplementary Table 5. ChIP-qPCR Primers

Gene Name	mST2 forward	mST2 reverse
Mouse ST2	5’-AAGGCACACCATAAGGCTGA-3’	5’-TCGTAGAGCTTGCCATCGTT-3’
