# Supplementary material for: ST2/IL-33 axis blockade inhibits regulatory T cell cytotoxicity towards CD8 T cells in the leukemic niche
Source: Nat Commun. 2025 Jul 21;16:6580. doi: 10.1038/s41467-025-61647-8 (PMC12279971; doi:10.1038/s41467-025-61647-8)
Supplement: Supplementary file 2 — Description of Additional Supplementary Files [file 41467_2025_61647_MOESM2_ESM.pdf]

Description of Additional Supplementary Files

Supplementary Data 1. Nanostring raw data of BM-derived WT T_{reg} cells and $Tbet^{-/-}$ T_{reg} cells sorted from sex- and age-matched normal naïve WT and $Tbet^{-/-}$ mice.
