# Supplementary material for: ST2/IL-33 axis blockade inhibits regulatory T cell cytotoxicity towards CD8 T cells in the leukemic niche
Source: Nat Commun. 2025 Jul 21;16:6580. doi: 10.1038/s41467-025-61647-8 (PMC12279971; doi:10.1038/s41467-025-61647-8)
Supplement: Supplementary file 4 — Reporting Summary [file 41467_2025_61647_MOESM4_ESM.pdf]

nature portfolio

Corresponding author(s): Sophie Paczesny

Last updated by author(s): Jun 12, 2025

Reporting Summary

Nature Portfolio wishes to improve the reproducibility of the work that we publish. This form provides structure for consistency and transparency in reporting. For further information on Nature Portfolio policies, see our Editorial Policies and the Editorial Policy Checklist.

Statistics

For all statistical analyses, confirm that the following items are present in the figure legend, table legend, main text, or Methods section.

n/a	Confirmed	
☐	☒	The exact sample size (*n*) for each experimental group/condition, given as a discrete number and unit of measurement
☐	☒	A statement on whether measurements were taken from distinct samples or whether the same sample was measured repeatedly
☐	☒	The statistical test(s) used AND whether they are one- or two-sided<br>*Only common tests should be described solely by name; describe more complex techniques in the Methods section.*
☐	☒	A description of all covariates tested
☐	☒	A description of any assumptions or corrections, such as tests of normality and adjustment for multiple comparisons
☐	☒	A full description of the statistical parameters including central tendency (e.g. means) or other basic estimates (e.g. regression coefficient) AND variation (e.g. standard deviation) or associated estimates of uncertainty (e.g. confidence intervals)
☐	☒	For null hypothesis testing, the test statistic (e.g. *F*, *t*, *r*) with confidence intervals, effect sizes, degrees of freedom and *P* value noted<br>*Give P values as exact values whenever suitable.*
☒	☐	For Bayesian analysis, information on the choice of priors and Markov chain Monte Carlo settings
☒	☐	For hierarchical and complex designs, identification of the appropriate level for tests and full reporting of outcomes
☐	☒	Estimates of effect sizes (e.g. Cohen's *d*, Pearson's *r*), indicating how they were calculated

Our web collection on statistics for biologists contains articles on many of the points above.

Software and code

Policy information about availability of computer code

Data collection	Attune NxT Flow Cytometer (Invitrogen), BD LSR II, BD LSRFortessa (X-20), BD FACSAria, Amnis Image StreamX MKII (Luminex), Illumina NovaSeq6000 softwares
Data analysis	GraphPad PRISM version 10.2.1(statistics/graphics), Adobe Illustrator (graphics), Flowjo 10.0.7 (Flow data analysis), IDES6.2 (Image flow data analysis), FastQC/MultiQC (Version 0.11.5,Sequencing quality), STAR(Version 2.5, Alignment), NGSUtils/bamutils (Version 0.5.9, Mapping quality), subread/featureCounts (Version 1.5.1, Read counting), edgeR (Version 3.28.1, Expression analysis), UCSC (Version mm10, Reference genome), UCSC (Version mm10/refGene, Genome annotation), FactoMineR(Version1.40, PCA plots), Clusterprofiler Package (Go analysis), Pheatmap Package (Heatmap), Chromium Single-Cell Instrument (10x Genomics, Pleasanton, CA, USA)Raw base call (BCL) files were analyzed using CellRanger (v7.0.0) 82. The “mkfastq” command was used to generate FASTQ files and the “count” command was used to generate raw gene – cell expression matrices. Ambient RNA contamination was inferred and removed using CellBender (v0.2.0) with standard parameters. Human Genome hg38 was used for the alignment and gencode.v42 was used for gene annotation and coordinates

For manuscripts utilizing custom algorithms or software that are central to the research but not yet described in published literature, software must be made available to editors and reviewers. We strongly encourage code deposition in a community repository (e.g. GitHub). See the Nature Portfolio guidelines for submitting code & software for further information.

April 2023

Data

Policy information about availability of data

All manuscripts must include a data availability statement. This statement should provide the following information, where applicable:

- Accession codes, unique identifiers, or web links for publicly available datasets
- A description of any restrictions on data availability
- For clinical datasets or third party data, please ensure that the statement adheres to our policy

Regulatory T cells bulk RNA sequencing data that support the findings of this study have been deposited in the GEO repository with the accession code GSE189688. Raw data is provided for all Figures and supplementary Figures; the patients' scRNA sequencing have been deposited in the Gene Expression Omnibus (GEO) under accession identification GSE279904

Research involving human participants, their data, or biological material

Policy information about studies with human participants or human data. See also policy information about sex, gender (identity/presentation), and sexual orientation and race, ethnicity and racism.

Reporting on sex and gender	AML patients' BM samples and information were collected after obtaining consent in accordance with institutional review board-approved studies at Fred Hutchinson Cancer Research Center (FHCRC), and the demographic data are presented in Table S1. Healthy human BM frozen cells (catalog: 2S-101D) were purchased from Lonza.<br>Since all patients were completely deidentified to the investigators and not collected for this specific study, the samples are considered human data but not human participants per NIH and IRB . Therefore, only a limited demographics including sex and age were provided (see Table S1)
Reporting on race, ethnicity, or other socially relevant groupings	See above, this is NA for the study as no human participants were involved.
Population characteristics	See Table S1
Recruitment	Recruitment was not part of this study; we received samples from the repository deidentiied.
Ethics oversight	AML patients' BM samples and information were collected after obtaining consent in accordance with institutional review board-approved studies at Fred Hutchinson Cancer Research Center (FHCRC)

Note that full information on the approval of the study protocol must also be provided in the manuscript.

Field-specific reporting

Please select the one below that is the best fit for your research. If you are not sure, read the appropriate sections before making your selection.

☒ Life sciences ☐ Behavioural & social sciences ☐ Ecological, evolutionary & environmental sciences

For a reference copy of the document with all sections, see nature.com/documents/nr-reporting-summary-flat.pdf

Life sciences study design

All studies must disclose on these points even when the disclosure is negative.

Sample size	Sample size was determined based on prior studies and literature in this field using similar experimental paradigms. For<br>the key experiments, some necessary pre-test were performed to determine the optimal sample size. Power calculation for survival in our typical leukemia/tumors experiments studies is shown ibelow. If p1 denotes mortality at the end of observation period (usually 60 days) in the animals KO for ST2 or treated with anti-ST2 and p2 in the WT animals, then the appropriate number of animals, over a range of values, needed to detect the difference with 80% power. The sample sizes needed for a detection of ≥ 40% survival difference between KO ST2/anti-ST2 treated group and the WT group with 80% power is 12 per group.
Data exclusions	All relevant data are shown. No data were excluded from the analysis and raw data.
Replication	All experiments of this study have been reproduced using the same experiment set-up with similar results. The number of the independent replications has been stated in the Figure Legends.
Randomization	All mice used here were randomly assigned to RNA-sequencing, leukemia survival monitoring, in vivo toxicity detection and PK/PD analysis.
Blinding	Researchers were not blind to tested groups. The researchers treating the mice were the same as those analyzing the data. The tested animals had to be clearly identified throughout<br>the study to prevent cross contamination in the cases of different allografts transplantation or different agents infusion. All statistics were

performed in an unbiased manner.

Reporting for specific materials, systems and methods

We require information from authors about some types of materials, experimental systems and methods used in many studies. Here, indicate whether each material, system or method listed is relevant to your study. If you are not sure if a list item applies to your research, read the appropriate section before selecting a response.

Materials & experimental systems

n/a	Involved in the study
☐	☒ Antibodies
☒	☐ Eukaryotic cell lines
☒	☐ Palaeontology and archaeology
☐	☒ Animals and other organisms
☒	☐ Clinical data
☒	☐ Dual use research of concern
☒	☐ Plants

Methods

n/a	Involved in the study
☒	☐ ChIP-seq
☐	☒ Flow cytometry
☒	☐ MRI-based neuroimaging

Antibodies

Antibodies used

1.Mouse antibosies for flow cytometry
Anti-mouse CD4-APC-eF780 (GK1.5), Cat. #47-0041-82, Lot: #2227586, eBioscience, 1:200 dilution;
Anti-mouse CD4-BV605 (RM4-5), Cat. #563151, Lot: #0314025, BD Bioscience, 1:200 dilution;
Anti-mouse CD8a-PE-Efluor610 (53-6.7), Cat. #61-0081-82, Lot. #2319702, eBioscience, 1:300 dilution;
Anti-mouse CD8a-BV786 (53-6.7), Cat. #563332, Lot. #9336120, BD Bioscience, 1:300 dilution;
Anti-mouse CD8b-PE(H35-17.2), Cat. #12-0083-82, Lot. #2112500, eBioscience, 1:200 dilution;
Anti-mouse ST2-PE(DJ8), Cat. #101001PE, Lot. #534563-A, Mdbioscience, 1:200 dilution;
Anti-mouse ST2-FITC(DJ8), Cat. #101001F, Lot. #S170404, Mdbioscience, 1:200 dilution;
Anti-mouse Foxp3-PE-Cy5.5 (FJK-16s), Cat. #35-5773-82, Lot. #2349256, eBioscience, 1:100 dilution;
Anti-mouse Foxp3-PercP-Cy5.5 (FJK-16s), Cat. #45-5773-82, Lot. #2283154, eBioscience, 1:100 dilution;
Anti-mouse Foxp3-PE-Cyanine7(FJK-16s), Cat. #25-5773-82, Lot. #2254250, eBioscience, 1:100 dilution;
Anti-mouse Foxp3-eF450 (FJK-16s), Cat. #48-5773-82, Lot. #1988692, eBioscience, 1:100 dilution;
Anti-mouse IFNγ-BV786 (XMG1.2), Cat. #563773, Lot. #0276624, BD Bioscience, 1:100 dilution;
Anti-mouse IFNγ-Alexa Fluor647 (XMG1.2), Cat. #557735, Lot. #9204800, BD pharmingen, 1:100 dilution;
Anti-mouse CD90.2-FITC (30-H12), Cat. #11-0903-85, Lot: #E00430-1630, eBioscience, 1:200 dilution;
Anti-mouse CD90.2-BV605 (30-H12), Cat. #105343, Lot: #B327974, Biolegend, 1:200 dilution;
Anti-mouse CD90.2-PE-Cyanine7 (30-H12), Cat. #25-0902-82, Lot: 4274383, eBioscience, 1:200 dilution;
Anti-mouse CD3-BV605 (17A2), Cat. #564009, Lot: #0342561, BD Bioscience, 1:200 dilution;
Anti-mouse CD3-BV711 (17A2), Cat. #67-0032-82, Lot: #2284076, eBioscience, 1:200 dilution;
Anti-mouse CD3e-BV650 (145-2C11), Cat. #564378, Lot: #9331240, BD Bioscience, 1:200 dilution;
Anti-mouse IL-17A-BV605 (TC11-18H10), Cat. #564169, Lot: #0247122, BD Bioscience, 1:100 dilution;
Anti-mouse IL-4-PE-Cyanine7((BVD6-24G2), Cat. #25-7042-42, Lot. # 1914366, eBioscience, 1:100 dilution;
Anti-mouse IL-4-APC (11B11), Cat. #17-7041-82, Lot. # E07365-1630, eBioscience, 1:100 dilution;
Anti-mouse IL-10-APC (JES5-16E3), Cat. #17-7101-82, Lot. # 1976255, eBioscience, 1:100 dilution;
Anti-mouse IL-10-BV711 (JES5-16E3), Cat. #564081, Lot. # 8256666, BD Bioscience, 1:100 dilution;
Anti-mouse IL-13-eFlour660 (eBio13A), Cat. #50-7133-82, Lot. # E16885-101, eBioscience, 1:100 dilution;
Anti-mouse IL-13-PercP-eFlour710 (eBio13A), Cat. #46-7133-82, Lot. # E16170-101, eBioscience, 1:100 dilution;
Anti-mouse LAP-PercP-eFlour710 (TW7-16B4), Cat. #46-9821-82, Lot. # 2093790, eBioscience, 1:100 dilution;
Anti-mouse Ki67-eFlour506 (SolA15), Cat. #69-5698-82, Lot. # 2305725, eBioscience, 1:100 dilution;
Anti-mouse Ki67-AF700 (SolA15), Cat. #56-5698-82, Lot. # 2261476, eBioscience, 1:100 dilution;
Anti-mouse KLRG1-APC(2F1), Cat. #17-5893-82, Lot. # 4323183, eBioscience, 1:100 dilution;
Anti-mouse KLRG1-SB780(2F1), Cat. #78-5893-82, Lot. # 1994844, eBioscience, 1:100 dilution;
Anti-mouse -Tbet-V450(O4-46), Cat. #561312, Lot. # 27524, BD Bioscience, 1:100 dilution;
Anti-mouse -Tbet-BV711(O4-46), Cat. #563320, Lot. # 1134162, BD Bioscience, 1:100 dilution;
Anti-mouse -Tbet-PE-Cyanine7(eBio4B10), Cat. #25-5825-82, Lot. # 4277988, eBioscience, 1:100 dilution;
Anti-mouse -TIM3-APC(8B.2C12), Cat. #17-5871-82, Lot. # 4271729, eBioscience, 1:100 dilution;
Anti-mouse -TIM3-BV786(5D12/TIM-3), Cat. #747621, Lot. # 0084759, BD Bioscience, 1:100 dilution;
Anti-mouse - LAG3-PE-Cyanine7(C9B7W), Cat. #25-2231-82, Lot. # 4284448, BD Bioscience, 1:100 dilution;
Anti-mouse - GITR-PercP-eFlour710(DTA-1), Cat. #46-5874-82, Lot. # 2016861, eBioscience, 1:100 dilution;
Anti-mouse - CTLA4-APC(UC10-4B9), Cat. #17-1522-82, Lot. # 2046231, eBioscience, 1:100 dilution;
Anti-mouse - TIGIT-BV711(1G9), Cat. #744214, Lot. # 8320751,BD Bioscience, 1:100 dilution;
Anti-mouse - TIGIT-eF660(GIGD7), Cat. #50-9501-82, Lot. # E15694-104,eBioscience, 1:100 dilution;
Anti-mouse - CD279(PD-1)-PercP-eFlour710(J43), Cat. #46-9985-82, Lot. # 2123831,eBioscience, 1:100 dilution;
Anti-mouse - CD279(PD-1)-BV786(RMP1-30), Cat. #748264, Lot. # 1349776, BD Bioscience, 1:100 dilution;

Anti-mouse - Granzyme A-APC,(GzA-3G8.5) Cat. #17-5831-82, Lot. # 2372941, eBioscience, 1:100 dilution;
Anti-mouse - Granzyme B-eF450,(NGZB) Cat. #48-8898-82, Lot. # 2096575, eBioscience, 1:100 dilution;
Anti-mouse - Granzyme B-PercP-eF710,(NGZB) Cat. #46-8898-82, Lot. # 2129921, eBioscience, 1:100 dilution;
Anti-mouse - CD45.1-APC-eF780,(A20) Cat. #47-0453-82, Lot. # E10196-1635, eBioscience, 1:200 dilution;
Anti-mouse - CD45-SB645,(104) Cat. #64-0451-82, Lot. # 2035975, eBioscience, 1:200 dilution;
Anti-mouse - F4/80-AF700,(BM8) Cat. #56-4801-82, Lot. # 2305719, eBioscience, 1:200 dilution;
Anti-mouse - CD107a-PE,(1D4B) Cat. #121612 Lot. # B279995, Biolegend, 1:200 dilution;
Anti-mouse - CD25-APC(PC61.5), Cat. #17-0251-82, Lot. # 2154040, eBioscience, 1:100 dilution;
Anti-mouse - CD127-BV650(A7R34), Cat. #135043, Lot. # B262432,Biolegend, 1:100 dilution;
Anti-mouse - Ly6G-APC(RB6-8C5), Cat. #17-5931-82, Lot. # E07334-1632,eBioscience, 1:200 dilution;
Anti-mouse - CD11b-eF450(M1/70), Cat. #48-0112-82, Lot. # 2198693,eBioscience, 1:100 dilution;
Anti-mouse - Ly6C-APC-Cy7(HK1.4), Cat. #47-5932-82, Lot. # 2209835,eBioscience, 1:200 dilution;
Anti-mouse - B220-FITC(RA3-6B2), Cat. #11-0452-85, Lot. # 4290821,eBioscience, 1:200 dilution;
Anti-mouse - Perforin-FITC(eBioOMAK-D), Cat. #11-9392-82, Lot. # 2321295,eBioscience, 1:200 dilution;
Anti-mouse - NK1.1-eF450(PK136), CatCell staining dyes for flow cytometry
2. Cell staining dyes for flow cytometry
FITC-Annexin V, Cat. #556419, Lot. # 0316912, BD Bioscience, 1:20 dilution;
Fixable Viability, Cat. #65-0866-14, Lot. # 2010926, eBioscience, 1:1000 dilution;
SYTOX-Blue, Cat. #S34857,Lot. # 1678820, Invitrogen, 1:1000 dilution;
CellTrace™ CFSE Cell Proliferation Kit, Cat. #S34554,Lot. # 1234547, Invitrogen,
3. Human antibodies for flow cytometry
Anti-human CD3-PE-eFluor610 (OKT3), Cat. #61-0037-42, Lot: #2016190, eBioscience, 1:50 dilution;
Anti-human CD4-V500 (RPA-T4), Cat. #560768, Lot: #28856, BD Bioscience, 1:50 dilution;
Anti-human CD8-AF700 (RPA-T8), Cat. #557945, Lot: #9094881, BD Bioscience, 1:50 dilution;
Anti-human IFNγ-PercP-Cyanine5.5(4S.B3), Cat. #45-7319-42, Lot: #E13640-104, eBioscience, 1:20 dilution;
Anti-human IFNγ-FITC (4S.B3), Cat. #11-7319-82, Lot: #1941241, eBioscience, 1:20 dilution;
Anti-human ST2-FITC(B4E6), Cat. #101002F, Lot. #S1912001, Mdbioscience, 1:20 dilution;
Anti-human Granzyme B-BV510(GB11), Cat. #563388, Lot. #7279671, BD Bioscience, 1:20 dilution;
Anti-human CD279(PD-1)-APC-eF780(eBioJ105), Cat. #47-2799-42, Lot: #4342449, eBioscience, 1:20 dilution;
Anti-human CD279(PD-1)-BV605(eh12.2), Cat. #563245, Lot: #8123980, BD Bioscience, 1:20 dilution;
Anti-human Ki67-PercP-eFlour710 (20Raj1), Cat. #46-5699-42, Lot. # E14340-107, eBioscience, 1:20 dilution;
Anti-human Foxp3-PE-eFlour610 (PCH101), Cat. #61-4776-42, Lot. #2288676, eBioscience, 1:20 dilution;
Anti-human KLRG1-BV711(2F1/KLRG1), Cat. #138427, Lot. # B294271, eBioscience, 1:20 dilution;
Anti-human -Tbet-PercP-Cyanine5.5(4B10), Cat. #45-5825-82, Lot. # 2094479, eBioscience, 1:20 dilution;. #48-5941-82, Lot. # E10940-1631,eBioscience, 1:100 dilution;

Validation

Commonly available antibodies, RNA and protein extraction kits have been used with established staining protocols. The following procedures will be utilized to ensure authentication of key resources:
1) Experiments will employ commercially available reagents purchased from reputable vendors that utilize stringent quality control measures for authentication (i.e. ebioscience, R&D, BD Bioscience, Miltenyi...).
2) Following receipt by the laboratory, the Technical Data Sheet and lot number of each reagent is logged and all manufacturer recommendations for storage, preparation and authentication are strictly followed to ensure stability and reproducible results.
3) The lot number of each resource (antibody, kits) is recorded upon receipt from vendor and its appropriate activity is independently authenticated by the laboratory prior to use and at regular intervals.
4) Experiments using authenticated resources are performed in triplicate utilizing reproducible assays (FACS, ELISA) that have first been validated by the laboratory.

Animals and other research organisms

Policy information about studies involving animals; ARRIVE guidelines recommended for reporting animal research, and Sex and Gender in Research

Laboratory animals

All mice used in this study were C57BL/6J background (or back-crossed to the C57BL/6J background) and purchased from the Jackson Laboratory, maintained under
specific pathogen-free conditions.
For the primary leukemia model, three different stages of leukemia mice (at around 4~5 weeks old) were sacrificed, and the bone marrow cells were directly analyzed on
flow cytometry.
For MLL-AF9 and DNMT3A/FLT3ITD transferred leukemia models, age and sex matched C57BL/6J mice (8~10 weeks old) were used for survival monitoring, therapeutic
antibody treatment and tumor growth assessments.
NOD.Cg-Prkdcscid Il2rgtm1wjl/SzJ (NSG) mice were used to construct humanized leukemic mice model (8~10 weeks old) and transferred with MOLM-14eGFP cells.

Wild animals

Was not selected

Reporting on sex	Multiple studies have shown that mice provide a very reliable and quantifiable model for studying leukemia models which ever sex is used.
Field-collected samples	Was not selected
Ethics oversight	All mouse experiments were done with the approval of Institutional Animal care and Use Committee of Indiana University and Medical University of South Carolina

Note that full information on the approval of the study protocol must also be provided in the manuscript.

Plants

Seed stocks	Was not selected
Novel plant genotypes	Was not selected
Authentication	Was not selected

Flow Cytometry

Plots

Confirm that:

- [x] The axis labels state the marker and fluorochrome used (e.g. CD4-FITC).
- [x] The axis scales are clearly visible. Include numbers along axes only for bottom left plot of group (a 'group' is an analysis of identical markers).
- [x] All plots are contour plots with outliers or pseudocolor plots.
- [x] A numerical value for number of cells or percentage (with statistics) is provided.

Methodology

Sample preparation	Single-cell suspensions were prepared from malignant or nonmalignant bone marrow niches or spleens, the dissociated cells were passed through 70 μm filters and pelleted. Red blood cells were completely lysed before surface staining with the flow antibodies. Gastraintestinal lamina propria lymphocytes isolation was performed as our previous study in GVHD. Briefly, the full length gastrointestines were flushed with phosphate buffered saline to remove fecal matter and mucus. Fragments (<0.5 cm) of intestines were digested in 10 ml of Dulbecco's modified Eagle's medium (DMEM) containing collagenase type B (2 mg/ml) (Roche), deoxyribonuclease I (10 mg/ml) (Roche), and 4% bovine serum albumin (Sigma) at 37°C with shaking for 90 min. The digested mixture was then diluted with 30 ml of plain DMEM, filtered through 70um strainers, and centrifuged at 800g for 10 min. The cell pellets were suspended in 4 ml of 70% Percoll (GE Healthcare), overlaid with 6 ml of 30% Percoll, and spun at 2000 rpm for 20 min at 4°C without braking. Enriched lymphocytes were collected from the interface
Instrument	Attune NxT Flow Cytometer (Invitrogen), BD LSR II, BD LSRFortessa (X-20), BD FACSAria, Amnis Image StreamX MKII (Luminex)
Software	Data were analyzed with FlowJo software Version 10.7.0 or IDEAS6.2
Cell population abundance	The purities of the sorted cells were more than 95%.
Gating strategy	See Figure S4 for a representative gating strategy. Based on the pattern of FSC-A/SSC-A, cells in the lymphocyte gate were used for analysis of T cell subsets. Singlets were gated according to the pattern of FSC-H vs.FSC-A. Positive populations were determined by the specific antibodies with compensations, which were distinct from negative populations. Bone marrow or spleen ST2 positive or negative regulatory T cells analysis was gated on singlets, Live/Dead dye-negative, CD45-positive, CD90.2/3-positive, CD4-positive, CD8-negative, Foxp3-positive, ST2-positive or ST2-negative events. Bone marrow or spleen IFNγ positive CD8+ T cells analysis was gated on singlets, Live/Dead dye-negative, CD45-positive, CD90.2/3-positive, CD8-positive, CD4-negative, IFNγ-positive events. Bone marrow or spleen IFNγ positive T conventional cells analysis was gated on singlets, Live/Dead Dye-negative, CD45-positive, TCRβ-positive, NK1.1-negative, CD4-positive, CD8-negative, Foxp3-negative, IFNγ-positive events.

Bone marrow or spleen IL-10/IL-4/IL-13/TGFbeta positive ST2 positive or negative regulatory T cells analysis was gated on singlets, Live/Dead dye-negative,

CD45-positive, CD90.2/3-positive, CD4-positive, CD8-negative, Foxp3-positive, ST2-positive or ST2-negative, IL-10 or IL-4 or IL-13 or TGFbeta positive events.

Bone marrow or spleen PD1/LAG3/TIM3/GITR positive CD8+T cells analysis was gated on singlets, Live/Dead dye-negative, CD45-positive, CD90.2/3-positive, CD4-negative, CD8-positive, PD1 or LAG3 or TIM3 or GITR positive events.

☒ Tick this box to confirm that a figure exemplifying the gating strategy is provided in the Supplementary Information.
